# The Epidemiological and Economic Burden of Vaccine-Preventable Respiratory Diseases Among Adults Aged 65 Years and Older in the Nordic Countries

**DOI:** 10.3390/vaccines14080703

**Published:** 2026-08-13

**Authors:** Nicoline Weinreich Reinstrup, Renée Hangaard Gidsel, Terho Heikkinen, Anne Margarita Dyrhol-Riise, Lars-Åke Levin, Lars Jørgen Østergaard, Lars Holger Ehlers

**Affiliations:** 1 Nordic Institute of Health Economics, 8000 Aarhus, Denmark; nicoline@reinstrup.dk (N.W.R.); renee.hangaard.olesen@rsyd.dk (R.H.G.); 2 Department of Pediatrics, University of Turku and Turku University Hospital, 20521 Turku, Finland; teheikki@utu.fi; 3 Department of Infectious Diseases, Oslo University Hospital, 0450 Oslo, Norway; a.m.d.riise@medisin.uio.no; 4 Institute of Clinical Medicine, University of Oslo, 0371 Oslo, Norway; 5 Department of Health Medicine and Caring Sciences, Linköping University, 58183 Linköping, Sweden; lars-ake.levin@liu.se; 6 Aarhus University Hospital, 8200 Aarhus, Denmark; larsoest@rm.dk

**Keywords:** burden of disease, economic burden, vaccination, vaccine-preventable respiratory diseases

## Abstract

**Background**: Despite the increasing availability of tailored vaccines, vaccine-preventable respiratory diseases (VPRDs), including influenza, respiratory syncytial virus (RSV), COVID-19, and pneumococcal disease (PD), continue to impose a significant health and economic burden. This study assessed the epidemiological and economic burden of VPRD among adults aged ≥65 across four Nordic countries (Denmark, Sweden, Norway, and Finland). **Methods**: Data on incidence, hospital admissions, and mortality were obtained from national statistics (week 21, 2024–week 20, 2025) and supplemented with peer-reviewed literature. A static model was developed to quantify epidemiological and economic burden associated with VPRD during the 2024/2025 winter season. **Results**: During the 2024/2025 season, VPRD resulted in approximately 830,000 symptomatic cases, 60,300 hospital admissions, and 7750 deaths across the Nordic countries. COVID-19 showed the highest estimated incidence and numbers of hospital admissions, followed by influenza, PD, and RSV. Mortality was comparable between COVID-19 and influenza, followed by PD and RSV. Annual healthcare costs were approximately €1132 million, with productivity losses constituting an additional 7%. **Conclusions**: Despite national vaccination efforts, VPRDs remain a major health and economic burden in the Nordic countries. Strengthening immunization programs through improved vaccine uptake, broader coverage, and use of advanced vaccines may contribute to further reducing this burden.

## 1. Introduction

Respiratory infections remain one of the leading causes of morbidity and mortality worldwide, with lower respiratory infections accounting for approximately 2.5 million deaths annually in the years preceding the COVID-19 pandemic [1,2,3]. Among vaccine-preventable respiratory diseases (VPRDs), influenza, pneumococcal disease (PD), respiratory syncytial virus (RSV), and, more recently, COVID-19, are the major contributors to the global disease burden [4,5,6,7,8]. These pathogens are associated with substantial rates of hospitalization and death, especially in older adults [4,9,10,11]. Their heightened vulnerability stems from age-related immunosenescence and the frequent presence of multiple comorbidities, both of which are associated with diminished immune function and vaccine responsiveness [4,9,10,11].

The epidemiological burden of VPRDs is mirrored by a large economic burden [12]. In general, elevated rates of VPRDs translate into considerable costs to the individual and society in terms of severe illness and deaths, leading to large healthcare costs and productivity losses. Seasonal surges of influenza and other respiratory viruses intensify these costs by straining healthcare systems and spiking hospital expenses, highlighting the importance of effective preventative measures [9,13,14,15,16].

Vaccination remains the most effective, cost-effective, and scalable intervention for preventing respiratory infections [7,9,17]. Nevertheless, despite the increasing availability of tailored vaccines, VPRD in older adults continues to impose a significant health and economic burden. The impact of respiratory infections has, to some extent, become normalized within the general population and is often perceived as an inevitable part of everyday life, with associated risks often downplayed as mainly relevant only to the most vulnerable groups [18,19,20]. In addition, underreporting and misclassification in health records obscure the true burden, as many cases go undiagnosed or unregistered. Consequently, policymakers may lack reliable data to accurately assess and prioritize preventive efforts [18,21]. In the public healthcare systems across the four Nordic countries in scope of this assessment (Denmark, Sweden, Norway, and Finland), decision-makers have historically adjusted vaccination strategies by expanding or narrowing target groups and adopting new vaccine types in line with shifts in disease patterns [22]. However, seasonal variations and uncertainty in the true burden of disease make these decisions challenging for public health authorities, especially in the context of limited healthcare budgets necessitating prioritization of preventative measures.

To help policymakers assess the burden of VPRDs, this study aims to estimate the current epidemiological and economic burden of influenza, COVID-19, PD, and RSV in adults 65 and older in the four Nordic countries and offer a better understanding to inform vaccination strategies and public health policy.

## 2. Materials and Methods

### 2.1. Study Design

The study consisted of two principal components. Initially, a structured literature search was conducted to identify studies and Health Technology Assessments (HTAs) that addressed the epidemiological and economic burden associated with COVID-19, influenza, RSV, and PD in older adult populations above 65 years of age. Subsequently, a static model was constructed utilizing national surveillance data and parameters extracted from the literature review to quantify incidence, hospital admissions, mortality, and costs for the four diseases in the four countries among older adults during the 2024/2025 season.

#### 2.1.1. Search Strategy

A structured search strategy was conducted in PubMed, CINAHL, Scopus, ProQuest, and Econlit [23]. All databases were searched for eligible literature on 27 March 2025. A block search was conducted with four search term groups pertaining to the themes of the elderly population, Nordic countries, respiratory diseases, and burden of illness. The search was limited to include literature published between 2014 and 2025, and was restricted to full-text literature in English, Danish, Swedish, Finnish, and Norwegian. The following were searched for HTAs: Medicintilskudsnævnet, SST, TLV, NT-rådet, SBU, NIPH, and Nye Metoder (Supplementary S2). All literature was aggregated using Rayyan [24]. Two independent researchers screened the title and abstract. Potentially relevant sources were retrieved and screened by one or more independent researchers. Furthermore, a review of publicly available national surveillance statistics from Nordic Health agencies was conducted.

##### Inclusion and Exclusion Criteria

Studies that reported epidemiological outcomes (incidence, hospital admissions, mortality) and/or economic outcomes (direct healthcare costs, indirect costs, cost-of-illness, unit costs, etc.) were included. Peer-reviewed publications, surveillance reports, or governmental documents from HTA databases were eligible. Studies were excluded if they lacked specific data on the mentioned epidemiological and economic outcomes for individuals aged ≥65 in the Nordic countries. All studies that were based on data collected during the COVID-19 pandemic (season 2019/2020, 2020/2021, and 2021/2022) were excluded due to the unique influence the pandemic posed on the spread of infectious diseases. Post-pandemic literature published after 2022 was prioritized, as these were considered more representative of usual disease patterns.

##### Data Extraction

Outcome measures from full-text articles, national surveillance systems, and HTA reports that met the inclusion criteria were extracted and assessed in relation to quality and consistency (Supplementary S3). The extracted data were prioritized according to evidence hierarchies that prioritize newer, comprehensive, and age-specific data sources reporting polymerase chain reaction (PCR) testing-confirmed cases [9].

#### 2.1.2. Statistics

A static model was developed in MS Excel 365 (Microsoft Corp., Redmond, WA, USA) [25] to summarize the annual epidemiological and economic burden associated with the four diseases during the 2024/2025 season based on findings from the systematic literature search and national surveillance data. Surveillance data from week 21, 2024 to week 20, 2025 were retrieved from each of the four countries (Supplementary S4–S9). In all estimations, PD was defined only as invasive pneumococcal disease (IPD) and pneumococcal pneumonia from serotypes that are vaccine-preventable, based on the 23-valent pneumococcal polysaccharide vaccine (PPV23).

##### Epidemiological Burden Estimation

Disease incidences in season 2024/25 (crude and rate per 100,000) were estimated by applying age-specific attack rates for influenza and multiplication factors (MFs) for RSV [14,20,26,27,28]. PD incidence and mortality were estimated based on incidence rates identified in the literature (Supplementary S5 for details) [29,30,31,32,33,34]. Incidence for COVID-19 was estimated under the assumption of the same ratio between disease incidence and hospital admissions as influenza. Hospital admissions were estimated based on admission rates for IPD and pneumonia admissions (Supplementary S5 for details) [29,30,31,32,33,34]. RSV admission numbers were adjusted using MFs [35,36]. Since the COVID-19 pandemic, systematic testing has been applied across all respiratory hospital settings in Denmark with a consistent diagnostic testing method for influenza, COVID-19, and RSV. Thus, Danish hospital data could be applied to create reference/calibration targets for admissions and deaths for each country by adjusting for country-specific population size, national vaccine uptake, and vaccine effectiveness (VE) depending on the current national vaccination programs [35]. Disease-specific mortality estimates were based on national surveillance data, literature-derived rates, adjustments for under-ascertainment, and cross-country extrapolation. For further information, see Supplementary Materials.

##### Economic Burden Estimation

The analytical framework incorporated societal costs and healthcare resources allocated to preventing and managing VPRDs. Healthcare resources include direct medical costs, which comprised inpatient visits (overnight hospitalization admission), outpatient visits (hospital visit without overnight admission), and general practitioner (GP) consultations. Vaccination program costs consisted of the cost of vaccines (estimations based on available prices at the 2025 price level) and vaccine administration. Societal costs reflect a human capital approach to productivity loss due to illness-related absenteeism. Productivity losses were applied only to the estimated proportion of the population in employment (see details in Supplementary Table S6.4). The cost calculator followed a micro-costing approach, where total costs were calculated by multiplying the number of events by the unit cost of the event [37]. All economic inputs were obtained from national Health Technology Assessment guidelines or peer-reviewed publications [38,39,40,41]. All costs were converted to 2025 values using country-specific consumer price indices [42,43,44,45].

##### Scenario Analysis

Five scenario analyses were conducted to demonstrate the effect of seasonal variations, pandemic disruptions, and uncertainties in the estimations [1,46,47]. Scenario 1 estimates the burden based on the 2023/2024 season. Scenarios 2 and 3 illustrate seasons with high and low influenza burden based on confidence intervals from Somes et al. [26], and Scenario 4 illustrates a higher RSV burden based on modelled estimates from Li et al. [48]. Scenario 5 illustrates the uncertainty in economic outcomes assuming list prices for COVID-19 vaccines [49].

## 3. Results

### 3.1. Literature Search

**Figure 1:**
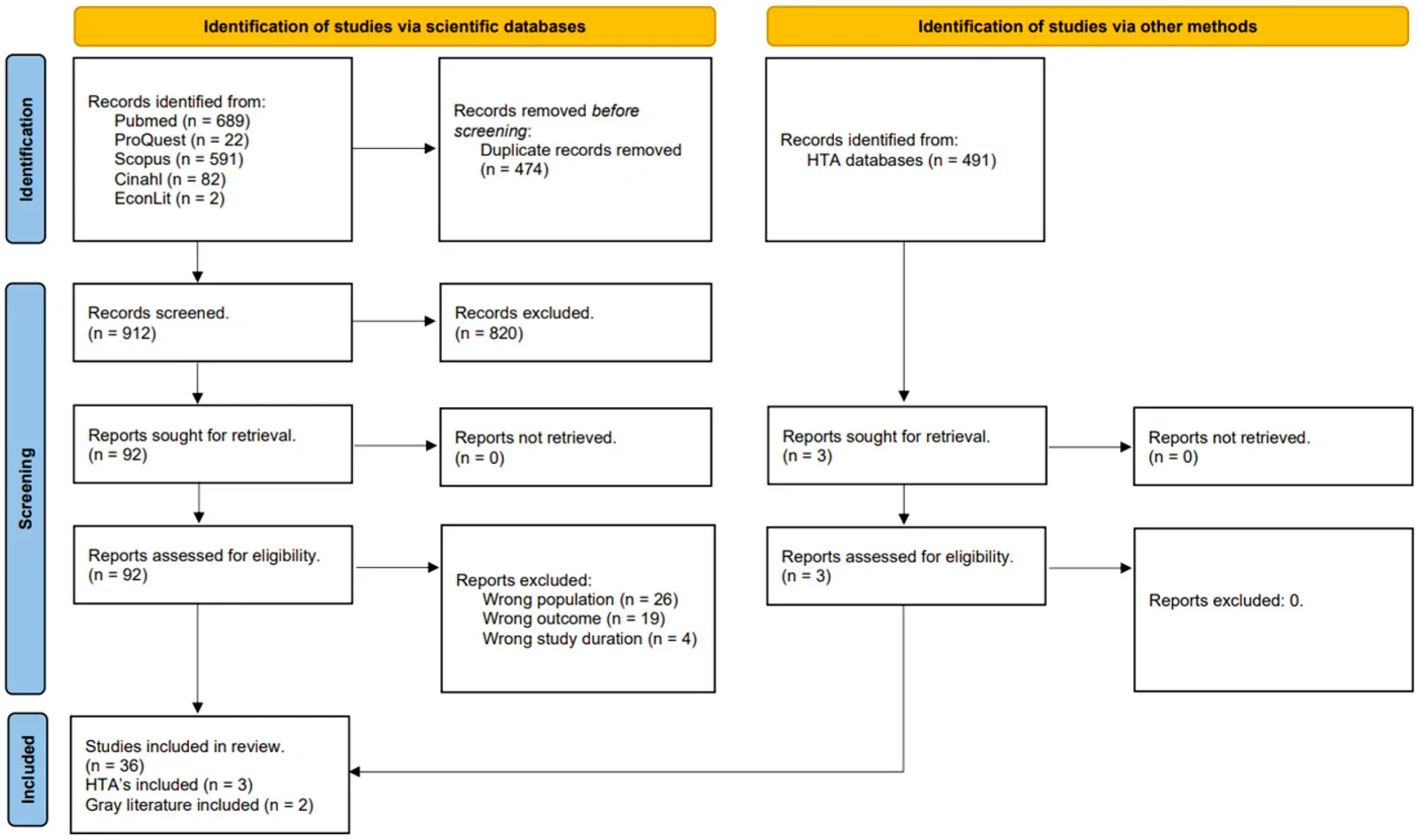
PRISMA flow chart.

A total of 912 unique records were identified through the structured literature search, and 98 articles were selected for full-text review. Forty-one met the inclusion criteria, with 13 reporting economic outcomes and 28 reporting epidemiological outcomes (Figure 1).

Across all countries, the highest number of studies were found for PD, and the lowest for COVID-19 (Supplementary S1). It should be noted that all studies that report on a subgroup of PD, e.g., IPD, pneumococcal meningitis, or pneumococcal pneumonia, were included. Furthermore, studies on the burden of COVID-19 were only included if they were based on post-pandemic data.

No study compared the burden of all diseases, with most studies focusing on a single disease within a single country. However, with regard to epidemiological studies, one study included three diseases (COVID-19, influenza, and RSV) [49], whereas three studies included two diseases: COVID-19 and influenza [45] and RSV and influenza [50,51]. Two studies included all Nordic countries [50,51], and two studies included three countries (Denmark, Norway, and Finland [21]; Denmark, Norway, and Sweden [22]). Only three epidemiological studies adjusted for underreporting, all of which modeled RSV hospitalizations [21,48,51]. Among the identified economic studies, none were cost-of-illness studies.

### 3.2. Epidemiological Burden

In total, there were 830,000 symptomatic cases of VPRD resulting in 60,300 hospital admissions, and 7750 deaths across the Nordic countries in the season 2024/25 (Table 1).

**Figure 2:**
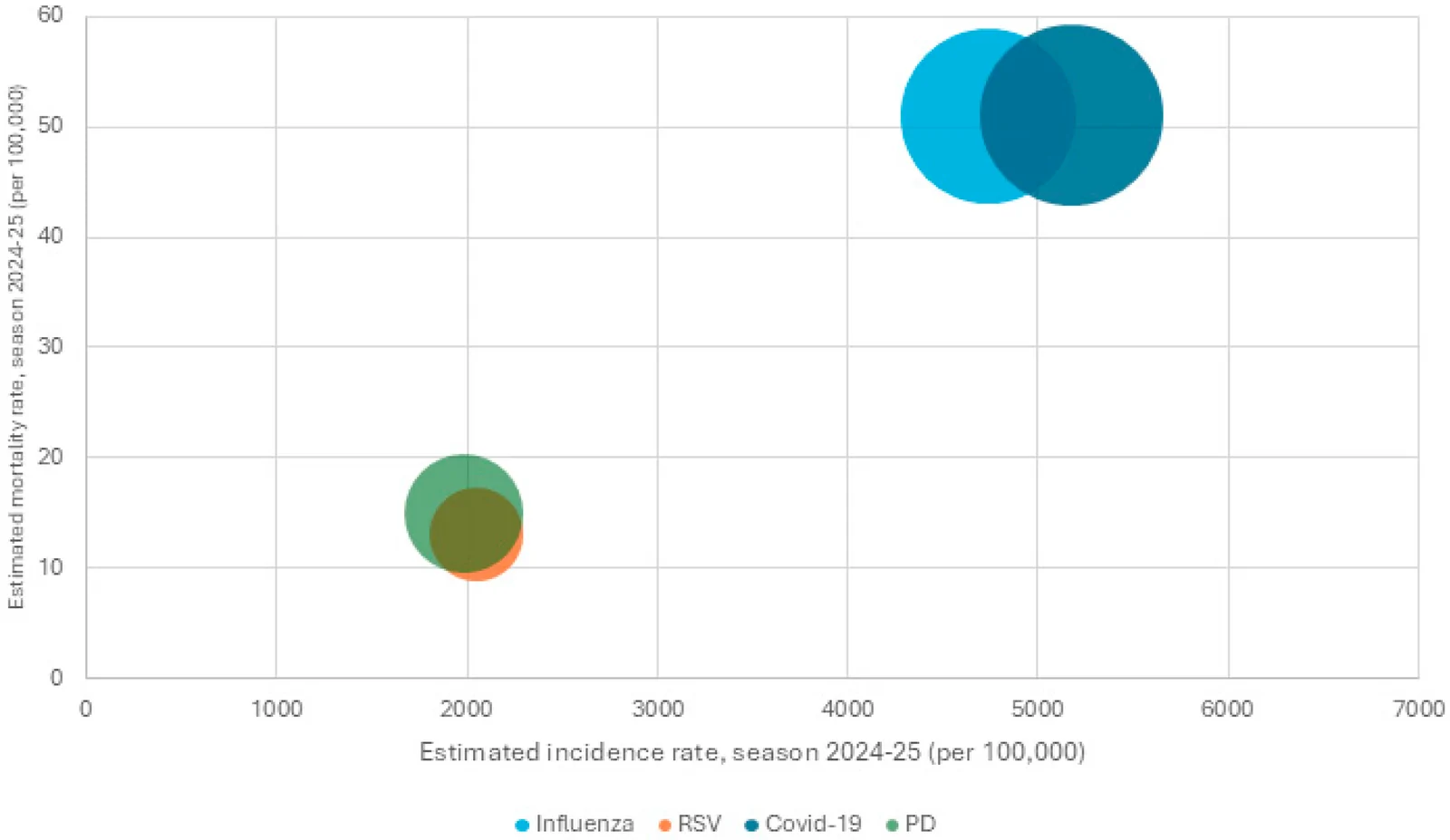
Bubble chart of the burden of COVID-19, influenza, PD, and RSV in terms of incidence, hospital admissions, and mortality collectively in the Nordic countries (Denmark, Sweden, Norway, and Finland) in the 2024/2025 season. The diameter of the bubble reflects the number of hospital admissions per 100,000 people.

The estimated incidence was 308,000 for COVID-19, 282,000 for influenza, 122,000 for RSV, and 118,000 for pneumococcal disease across all Nordic countries (Figure 2). Hospital admissions were 23,000 for COVID-19, 21,200 for influenza, 9700 for pneumococcal disease, and 6200 for RSV. Mortality was 3050 for influenza, 3010 for COVID-19, 920 for pneumococcal disease, and 770 for RSV. The epidemiological burden was similar across all Nordic countries, except for Sweden, where influenza cases and hospital admissions were estimated to be higher than for COVID-19. This difference potentially reflects variations in vaccination programs; for example, Denmark having a higher coverage rate of enhanced influenza vaccines.

COVID-19 posed a significantly greater burden, with roughly twice as many cases, admissions, and deaths in the season 2023/2024 as in the season 2024/2025 (Scenario 1). The importance of high vs. low influenza season showcases the impact of seasonal variation, as a high influenza season would constitute a much larger burden than COVID-19 (Scenario 2). For comparative purposes, a low influenza season was estimated in Scenario 3. Different methods for adjusting for underestimation in RSV demonstrated that RSV may potentially pose a larger burden than PD (Scenario 4). (All scenario analysis results are shown in Supplementary S7–S9.)

### 3.3. Economic Burden

The total societal costs of the VPRDs in the season 2024/2025 across the Nordics were €1213 million (Table 2). Costs were €189 million in Denmark, €580 million in Sweden, €204 million in Norway, and €241 million in Finland.

**Figure 3:**
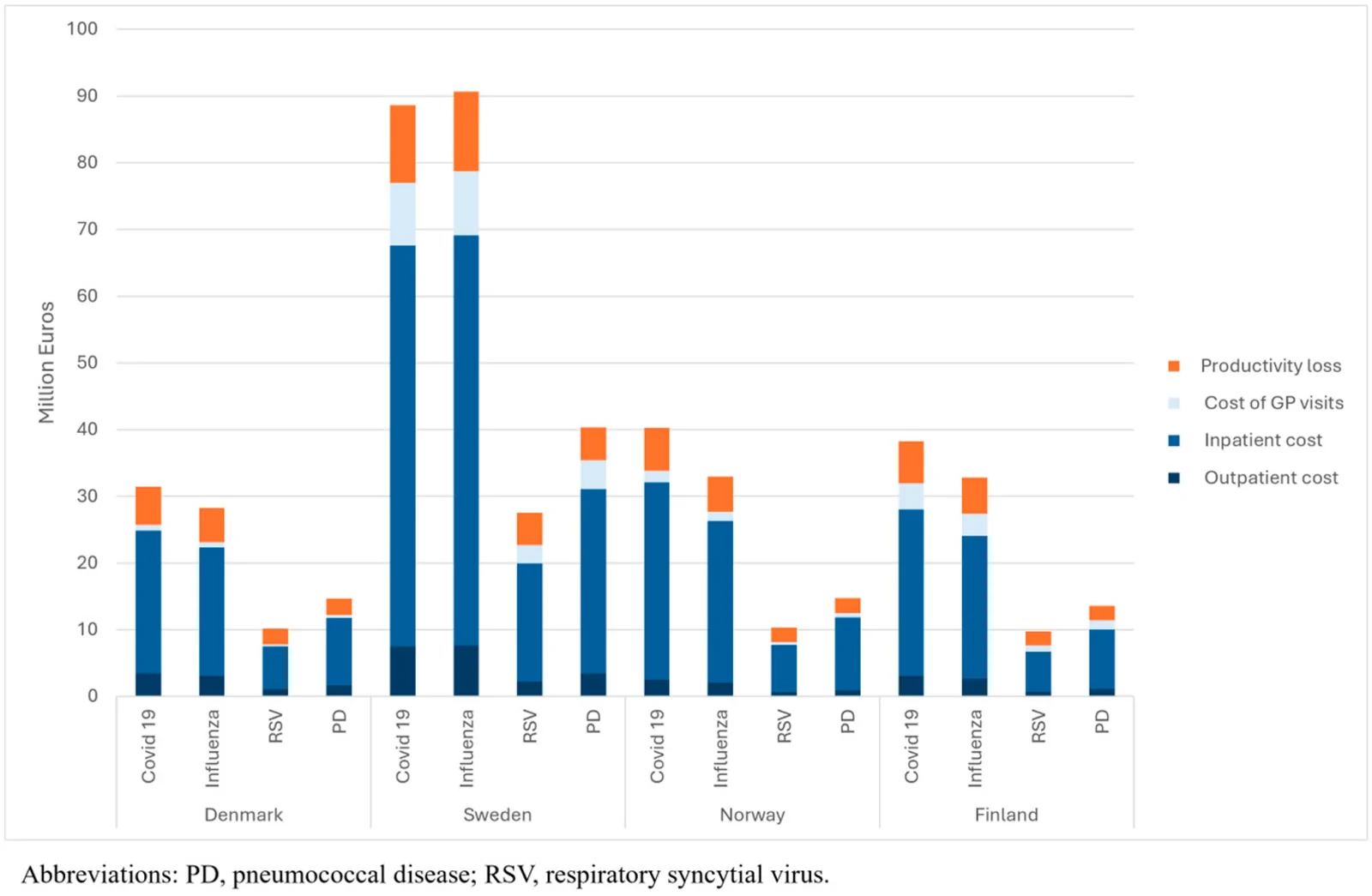
Economic burden estimates of COVID-19, influenza, RSV, and PD across the Nordic countries in the season 2024/2025, in million euros.

Healthcare costs were 93% of total costs, due to the low number of individuals aged 65 and older participating in the workforce (Figure 3).

**Figure 4:**
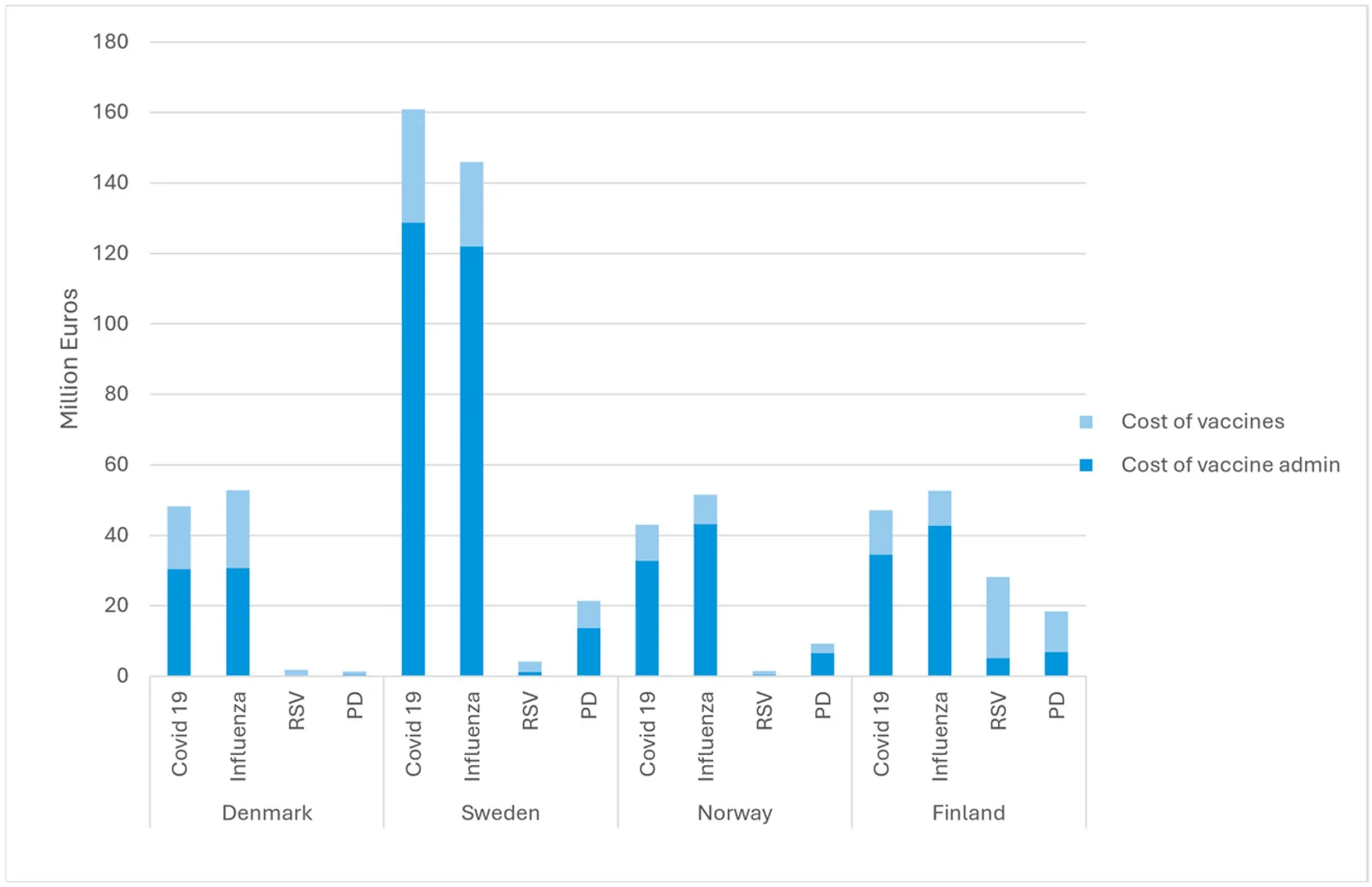
Cost of vaccines and vaccine administration for COVID-19, influenza, RSV, and PD across the Nordic countries in the season 2024/2025, in million euros.

Vaccine administration was the largest single cost driver, accounting for approximately €500 million (41% of total societal costs) (Figure 4).

Inpatient care represented the second largest component, with costs estimated at €357 million (29%). Vaccine acquisition costs across all vaccine-preventable respiratory diseases amounted to €189 million, corresponding to 16% of total societal costs. Productivity losses due to illness-related absenteeism were estimated at €81 million (7%), while outpatient visits (€44 million; 4%) and GP visits (€42 million; 3%) contributed smaller proportions of the overall burden (Supplementary Figures S8.1 and S9.1 and Tables S8.1 and S9.1 for further details).

Scenario 1 showed a substantially higher epidemiological burden of COVID-19 during the 2023/2024 season. Variations in influenza incidence, as modeled in high and low influenza season scenarios, resulted in 10% greater and 6% lower total costs, respectively (Scenarios 2 and 3), while assuming greater underestimation of RSV resulted in a 35% increase in total costs for RSV across all countries (Scenario 4). Assuming no discount on COVID-19 vaccines at official list prices resulted in a 53% increase in the total cost burden of COVID-19 (Scenario 5).

## 4. Discussion

The findings of this study indicate that older adults across the Nordic countries face a substantial cumulative epidemiological burden, especially from COVID-19 and influenza, while PD and RSV constituted a relatively lower burden in the season 2024/2025. The economic burden was in line with the epidemiologic burden, with COVID-19 and influenza as the main contributors; still, RSV and PD burden remain non-negligible. These findings highlight the importance of national multivalent vaccination strategies.

To the best of our knowledge, this investigation is the first study within the Nordic countries to examine the cumulative burden of these four diseases together. A notable strength of this study lies in its comprehensive and systematic review, including both scientific articles, HTA reports, and national surveillance data. A key limitation of the study is the difficulty of comparing epidemiological data across countries and pathogens. Publicly available statistics on VPRDs are generally affected by underdiagnosis and underreporting, particularly evident for PD, where testing for and reporting Streptococcus pneumoniae is typically limited to blood or cerebrospinal fluid cultures, leaving most non-invasive cases unreported [52,53,54,55]. In addition, PD was restricted to IPD and pneumococcal pneumonia caused by serotypes covered by PPV23. The estimates therefore do not capture the total burden of pneumococcal disease and may underestimate disease caused by non-PPV23 serotypes and non-invasive pneumococcal infections that are not microbiologically confirmed. Uncertainty is further compounded by differences in case registration and diagnostic coding across countries, with some data based on laboratory confirmation and others on ICD-10 codes [9,21,55]. Diagnostic practices also vary across diseases, age groups, and national contexts; for instance, Norwegian registry data (2020–2024) show RSV PCR testing in only 53% of hospitalized adults, compared with 73% for influenza and 82% for COVID-19, and below 50% in adults aged ≥65 years [56,57,58]. This could be due to the use of point-of-care tests for influenza and COVID-19. Additional uncertainty arises from differing methods for adjusting underestimation (21). For RSV and PD, hospitalization and incidence rates vary widely depending on case definition and data source [19,21,29,36,51,53,59,60,61,62,63,64,65]. Temporal variability further complicates interpretation, as respiratory infections show strong interannual and post-pandemic fluctuations, with off-season RSV and influenza epidemics after 2022 linked to waning population immunity and overlapping circulation with COVID-19 [9,35,36,46,56,57,66,67]. To address these uncertainties, we sought to account for variability in epidemiological estimates through scenario-based analyses.

Our economic estimates were generally in line with findings in other studies [9,50,51]. Cross-country differences were driven primarily by population size as well as variations in vaccination programs and coverage rates. Sweden and Finland also incurred higher administration costs. However, the uncertainties inherent in epidemiological burden estimates have implications for economic studies, as assumptions about incidence, hospitalization, and mortality rates serve as model inputs. Li et al. [61] showed that the choice of burden estimation method (ICD-codes, laboratory-confirmed cases, or time-series models) was an influential factor affecting the predicted cost-effectiveness of RSV vaccination. Consequently, the wide range of disease burden estimates found in the literature translates into uncertainty in estimates of the economic burden [29,61,62]. Finally, there is substantial uncertainty in the cost estimates for vaccines, where we have applied publicly available list prices. Confidential discounts may have reduced the cost to public payers significantly, especially for COVID-19, where pandemic EU contracting and advance purchase agreements may have resulted in substantially lower prices than those reflected in official list prices. This underlines the importance of the sensitivity analyses (Supplementary S7–S9).

The included costs were limited to direct health sector costs and productivity loss, omitting costs such as over-the-counter medicine and patient transportation, and mortality-related costs. Health consequences and financial burden can extend beyond the acute phase of infection (e.g., long-term post-infection sequelae costs), not captured in our estimations, suggesting that the total economic burden reported is likely conservative [9,68]. Finally, our estimates of hospitalizations may be conservative for influenza, as other international data from the last flu season showed that hospitalization rates were much higher for influenza compared to COVID-19 [69].

Our study may have different health-policy implications for each Nordic country. Overall, the findings suggest that despite existing vaccination programs, adults aged 65 years and older continue to experience a substantial health and economic burden from vaccine-preventable respiratory diseases. This highlights the need to strengthen national recommendations, continuing to encourage the uptake of enhanced (high-dose or adjuvanted) influenza vaccines, while integrating RSV and updated pneumococcal conjugate vaccines into routine schedules, and maintaining COVID-19 booster coverage. A coordinated strategy could optimize resource use, reduce hospitalizations, and support long-term health system sustainability among the aging populations in the Nordic countries.

Future research should prioritize establishing best-practice methods to estimate the hidden burden of VPRDs and making such information available annually for public health research and policy. Furthermore, research should undertake detailed cost-effectiveness studies in the Nordic context, as this is lacking for all four diseases, to provide policymakers with a robust foundation for priority setting and to ensure that policy decisions are informed by both clinical outcomes and economic considerations.

## 5. Conclusions

Despite national vaccination efforts, VPRDs remain a major health and economic burden in the Nordic countries. Strengthening immunization programs through improved uptake, broader coverage, and use of advanced vaccines may contribute to further reducing this burden.

## Figures and Tables

**Table 1:** Epidemiological burden estimates of COVID-19, influenza, RSV, and PD across the Nordic countries in the season 2024/2025.

	COVID-19		Influenza		RSV		PD	
	Estimated Crude (N)	Estimated Rate (per 100,000)	Estimated Crude (N)	Estimated Rate (per 100,000)	Estimated Crude (N)	Estimated Rate (per 100,000)	Estimated Crude (N)	Estimated Rate (per 100,000)
Denmark								
Incidence	58,000	4639	52,000	4159	26,000	2080	25,000	2000
Admissions	4400	352	3900	312	1300	104	2100	168
Mortality	470	38	570	46	160	13	200	16
Sweden								
Incidence	110,000	4760	112,000	4846	47,000	2034	46,000	1990
Admissions	8300	359	8500	368	2400	104	3800	164
Mortality	1410	61	1210	52	300	13	370	16
Norway								
Incidence	62,000	5847	51,000	4810	22,000	2075	21,000	1980
Admissions	4600	434	3800	358	1100	104	1700	160
Mortality	500	47	550	52	140	13	160	15
Finland								
Incidence	78,000	5870	67,000	5042	27,000	2032	26,000	1957
Admissions	5900	444	5000	376	1400	105	2100	158
Mortality	630	47	720	54	170	13	190	14
Total Nordics								
Incidence	308,000	5176	282,000	4739	122,000	2050	118,000	1983
Admissions	23,200	390	21,200	356	6200	104	9700	163
Mortality	3010	51	3050	51	770	13	920	15

**Table 2:** Economic burden estimates of COVID-19, influenza, RSV, and PD across the Nordic countries in the season 2024/2025.

	COVID-19	Influenza	RSV	PD
**Denmark**				
Total Healthcare Costs	73,920,000	75,900,000	9,540,000	13,580,000
Cost of vaccines	17,880,000	22,000,000	1,470,000	920,000
Cost of vaccine admin	30,340,000	30,780,000	290,000	450,000
Cost of GP visits	870,000	780,000	260,000	410,000
Outpatient cost	3,470,000	3,120,000	1,050,000	1,650,000
Inpatient cost	21,370,000	19,210,000	6,470,000	10,150,000
Total Societal Costs	79,670,000	81,070,000	11,970,000	16,070,000
Productivity loss	5,750,000	5,170,000	2,430,000	2,490,000
**Sweden**				
Total Healthcare Costs	237,960,000	224,710,000	26,920,000	56,810,000
Cost of vaccines	32,160,000	23,990,000	2,970,000	7,640,000
Cost of vaccine admin	128,780,000	121,980,000	1,240,000	13,740,000
Cost of GP visits	9,430,000	9,650,000	2,780,000	4,340,000
Outpatient cost	7,470,000	7,640,000	2,200,000	3,440,000
Inpatient cost	60,110,000	61,460,000	17,730,000	27,660,000
Total Societal Costs	249,610,000	236,620,000	31,730,000	61,730,000
Productivity loss	11,650,000	11,910,000	4,800,000	4,920,000
**Norway**				
Total Healthcare Costs	76,890,000	79,300,000	9,760,000	21,700,000
Cost of vaccines	10,250,000	8,480,000	1,140,000	2,730,000
Cost of vaccine admin	32,830,000	43,110,000	450,000	6,490,000
Cost of GP visits	1,740,000	1,420,000	420,000	640,000
Outpatient cost	2,540,000	2,080,000	610,000	940,000
Inpatient cost	29,530,000	24,200,000	7,130,000	10,900,000
Total Societal Costs	83,350,000	84,580,000	11,930,000	23,920,000
Productivity loss	6,460,000	5,280,000	2,180,000	2,230,000
**Finland**				
Total Healthcare Costs	79,000,000	80,010,000	35,830,000	29,900,000
Cost of vaccines	12,600,000	9,890,000	23,030,000	11,560,000
Cost of vaccine admin	34,490,000	42,740,000	5,150,000	6,910,000
Cost of GP visits	3,910,000	3,350,000	940,000	1,400,000
Outpatient cost	3,100,000	2,660,000	740,000	1,110,000
Inpatient cost	24,910,000	21,370,000	5,970,000	8,920,000
Total Societal Costs	85,300,000	85,410,000	37,940,000	32,030,000
Productivity loss	6,300,000	5,410,000	2,110,000	2,140,000
**Nordic countries**				
Total Healthcare Costs	467,770,000	459,920,000	82,050,000	121,990,000
Cost of vaccines	72,890,000	64,360,000	28,610,000	22,850,000
Cost of vaccine admin	226,440,000	238,610,000	7,130,000	27,590,000
Cost of GP visits	15,950,000	15,200,000	4,400,000	6,790,000
Outpatient cost	16,580,000	15,500,000	4,600,000	7,140,000
Inpatient cost	135,920,000	126,240,000	37,300,000	57,630,000
Total Societal Costs	497,930,000	487,680,000	93,570,000	133,750,000
Productivity loss	30,160,000	27,770,000	11,520,000	11,780,000

## Data Availability

The original contributions presented in this study are included in the article/Supplementary Materials. Further inquiries can be directed to the corresponding author.

## Supplementary Materials

The following supporting information can be downloaded at https://www.mdpi.com/article/10.3390/vaccines14080703/s1, S1: Systematic literature search outcomes; S2: National surveillance sources; S3: Extracted literature; S4: Vaccination programs; S5: Description of statistical methods; S6: Input tables; S7: Overview of scenario analyses; S8: Epidemiological burden results; S9: Economic burden Results; S10: References (supplementary).

## Abbreviations

The following abbreviations are used in this manuscript:

COVID-19	Coronavirus disease 2019
GP	General practitioner
HTA	Health Technology Assessment
ICD-10	International Classification of Diseases, 10th Revision
IPD	Invasive pneumococcal disease
MF	Multiplication factor
PCR	Polymerase chain reaction
PD	Pneumococcal disease
PPV23	23-valent pneumococcal polysaccharide vaccine
RSV	Respiratory syncytial virus
VE	Vaccine effectiveness
VPRD	Vaccine-preventable respiratory disease
