# Supplementary material for: The Epidemiological and Economic Burden of Vaccine-Preventable Respiratory Diseases Among Adults Aged 65 Years and Older in the Nordic Countries
Source: Vaccines (Basel). 2026 Aug 13;14(8):703. doi: 10.3390/vaccines14080703 (PMC13517237; doi:10.3390/vaccines14080703)
Supplement: Supplementary file 1 [file vaccines-14-00703-s001.zip › vaccines-4444909-supplementary.pdf]

Supplementary Materials

Contents

S1 Systematic literature search outcomes 2
S2 National surveillance sources 3
S3 Extracted literature 4
S4 Vaccination programs 6
S5 Description of statistical methods 7
S6 Input tables 12
S7 Overview of scenario analyses 16
S8 Epidemiological burden results 16
S9 Economic burden Results 25
S10 References (supplementary) 40

S1 Systematic literature search outcomes

Figure S1

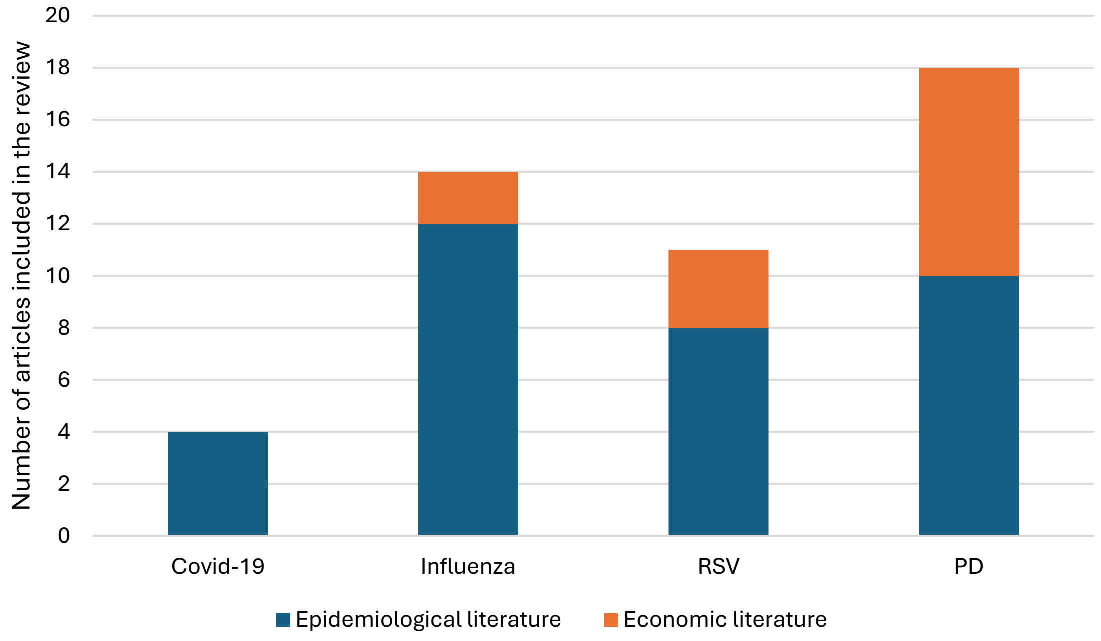

PD, pneumococcal disease; RSV, respiratory syncytial virus.

S2 National surveillance sources

Table S2: Overview of National and HTA agencies included in the literature search.

Country	Source
Denmark	Statens Serum Institut (ssi.dk)<br>Danmarks statistik (sst.dk)
Sweden	Folkhalsomyndigheten (Folkhalsomyndigheten.se)<br>Socialstyrelsen (socialstyrelsen.se)
Norway	Folkehelseinstituttet (fhi.no)
Finland	Terveyden ja hyvinvoinnin laitos(thl.fi)<br>Statistics Finland (stat.fi)
Other	European Respiratory Virus Surveillance Summary (erviss.org)<br>World Health Organization (WHO.int)<br>European Centre for Disease Prevention and Control (ecdc.europa.eu)

S3 Extracted literature

Table S3: Overview of studies identified through literature search and search in HTA databases based on outcomes

	COVID-19	Influenza	RSV	PD
Incidence	Poukka et al. 2024 [1]	Jacob et al. 2023 [2]<br>Hauge et al. 2018 [3]<br>Emborg et al. 2023 [4]<br>Alvarez et al. 2023 [5]<br>Baum et al. 2021 [6]	Li et al. 2025 [7]<br>TLV 2023a [8]<br>TLV 2023b [9]	Malene et al. 2023 [10]<br>Steens et al. 2014 [11]<br>Lyngstad et al. 2022 [12]<br>Nymark et al. 2022 [13]<br>Palmborg et al. 2023 [14]<br>Winje et al. 2021 [15]<br>Sevilla et al. 2019 [16]<br>Olsen et al. 2022 [17]<br>Birck et al. 2021 [18]<br>Wolff et al. 2020 [19]<br>Zarabi et al. 2023 [20]<br>Naucler et al. 2019 [21]<br>Naucler et al. 2017 [22]<br>Backhaus et al. 2016 [23]<br>Pugh et al. 2020 [24]<br>Polkowska et al. 2021 [25]<br>Nuorti et al. 2022 [26]
Mortality	Seppala et al. 2025 [27]<br>Bager et al. 2025 [28]<br>Poukka et al. 2024 [1]	Seppala et al. 2025 [27]<br>Hauge et al. 2018 [3]<br>Nielsen et al. 2018 [29]<br>Jacob et al. 2023 [2]<br>Bager et al. 2025 [28]<br>Nersesjan et al. 2020 [30]<br>Alvarez et al. 2023 [5]<br>SST 2021 [31]	Li et al. 2025 [7]<br>TLV 2023a [8]<br>TLV 2023b [9]<br>Urchueguía-Fornes et al. 2024 [32]	Malene et al. 2023 [10]<br>Sevilla et al. 2019 [16]<br>Olsen et al. 2022 [17]<br>Lopez-Lacort et al. 2024 [33]<br>Birck et al. 2021 [18]<br>Nuorti et al. 2022 [26]
Hospitalizations	Seppala et al. 2024 [34]<br>Seppala et al. 2025 [27]<br>Bager et al. 2025 [28]<br>Poukka et al. 2024 [1]	Seppala et al. 2024 [34]<br>Debes et al. 2021 [35]<br>Seppala et al. 2025 [27]<br>Jacob et al. 2023 [2]<br>Hauge et al. 2018 [3]<br>Emborg et al. 2023 [4]<br>Bager et al. 2025 [28]<br>SST 2021 [31]<br>Auvinen et al. 2021 [36]<br>Alvarez et al. 2023 [5]<br>Halme et al. 2022[37]<br>Baum et al. 2021 [6]	Seppala et al. 2025 [27]<br>Debes et al. 2021 [35]<br>Osei-Yeboah et al. 2024 [38]<br>Osei-Yeboah et al. 2023 [39]<br>Johannesen et al. 2022 [40]<br>Johannesen et al. 2025 [41]<br>Li et al. 2025 [7]<br>Urchueguía-Fornes et al. 2024 [42]	Lyngstad et al. 2022 [12]<br>Lopez-Lacort et al. 2024 [33]<br>Wolff et al. 2019 [19]<br>Zarabi et al. 2023 [20]<br>Nymark et al. 2022 [13]<br>Olsen et al. 2022 [17]<br>Birck et al. 2021 [18]

			Auvinen et al. 2021 [36]	
Economic studies		Jacob et al. 2023 [2]<br>Alverez et al. 2023 [5]	Li et al. 2025 [7]<br>TLV 2023a [8]<br>TLV 2023b [9]	Sevilla et al. 2019 [16]<br>Birck et al. 2021 [18]<br>Wolff et al. 2020 [19]<br>Zarabi et al. 2023 [20]<br>Lyngstad et al. 2022 [12]<br>Malene et al. 2023 [10]<br>Olsen et al. 2022 [17]<br>Nymark et al. 2022 [13]

PD, pneumococcal disease; RSV, respiratory syncytial virus.

S4 Vaccination programs

Table S4: Overview of public vaccination programs in the Nordic countries in season 2024/2025, advised for people 65+ by advisory committees or health authorities.

Country	Influenza	COVID-19	PD	RSV
**Denmark**	Recommended for 65+ and enhanced vaccines for 70+	Recommended for 65+ annually	Recommended for risk groups	Recommended for risk groups
**Sweden**	Recommended for 65+ annually and enhanced vaccines for nursing home residents	Recommended for 65+ annually	Recommended the year you turn 75 and risk groups	Recommended the year you turn 75 and risk groups
**Norway**	Recommended for 65+ and nursing home residents annually. Enhanced vaccines preferred for the most vulnerable older adults	Recommended for 65+ annually	Recommended for 65+ and risk groups	Not included in the public vaccination program
**Finland**	Recommended for 65+, and enhanced vaccines for 85+	Recommended for 65+ annually	Recommended for risk groups 65+	Not included in the public vaccination program

PD, pneumococcal disease; RSV, respiratory syncytial virus.

S5 Description of statistical methods

Overall modelling framework

Overview 1 provides an overview of the analytical framework used to estimate the epidemiological and economic burden of vaccine-preventable respiratory diseases (VPRDs) among adults aged 65 years and older in the Nordic countries.

Overview 1. Overall analytical framework used to estimate the epidemiological and economic burden of vaccine-preventable respiratory diseases among adults aged ≥65 years in the Nordic countries.

1. Input
Literature
Population data
Surveillance data
Vaccination programs, uptake and effectiveness
Expert validation
Unit costs
↓
2. Integrated Epidemiological Model
Danish reference estimates
Disease-specific modelling (influenza, covid-19, RSV, pneumococcal)
Country-specific extrapolation & adaptation
↓
3. Epidemiological Burden
Symptomatic cases
Hospital admissions
Deaths
↓
4. Economic Burden
Healthcare costs (vaccines, administration, GP, outpatient, inpatient)
Societal costs (productivity losses)
↓
5. Sensitivity analyses
Seasonal variation, under-ascertainment, vaccine prices

The framework consists of two sequential modelling components. First, an epidemiological model was developed to estimate the annual burden of COVID-19,

influenza, respiratory syncytial virus (RSV), and pneumococcal disease (PD) in terms of symptomatic cases, hospital admissions, and deaths.

Because the availability and quality of surveillance data differed between diseases, disease-specific modelling approaches were applied. Influenza estimates were primarily based on surveillance data combined with vaccination-adjusted attack rates, COVID-19 estimates were derived using national surveillance data and estimated relationships between symptomatic infections and hospitalisations, RSV estimates incorporated adjustments for under-ascertainment and healthcare-seeking behaviour (because RSV, unlike COVID-19 and influenza, is not widely included in national vaccination programs for elderly), and pneumococcal disease estimates combined invasive pneumococcal disease and pneumococcal pneumonia using serotype-specific information where available.

The model integrated multiple evidence sources, including national surveillance data, published literature, demographic data, vaccination programme characteristics, vaccine uptake, vaccine effectiveness, and disease-specific modelling assumptions. Danish surveillance data were used as a key reference source where they provided the most complete and robust evidence, while country-specific data were incorporated whenever available and considered sufficiently reliable. In the post pandemic period in Denmark, laboratory-confirmed testing of all patients presenting themselves at healthcare settings with symptoms of COVID-19, influenza, and/or RSV were normal practice. The resulting epidemiological estimates represent the best possible country-specific burden estimates for each of the four Nordic countries based on publicly available data and a common analytical framework.

The epidemiological estimates were subsequently used as inputs to the economic model. A micro-costing approach was applied to estimate direct healthcare costs associated with national specific information on vaccination programs, general practitioner consultations, outpatient visits, and hospital admissions, together with indirect costs related to productivity losses. Country-specific unit costs were obtained from official national sources and published literature whenever available. The framework integrates epidemiological evidence and economic modelling into a single analytical structure, allowing estimation of both the epidemiological burden and the societal economic burden associated with VPRDs across the Nordic countries. The individual components of the model are described in detail in the following sections.

Epidemiological inputs

Disease Incidence

Influenza incidence is estimated in accordance with a method frequently used in the health economic literature, including De Fougerolles et al. [43], Jacob et al. [2] and Alvarez et al. [5]. Here, an attack rate for symptomatic cases among individuals aged 65 and above from the meta-analysis by Somes et al. [44] is used to estimate incidence. This estimate is adjusted for vaccine coverage and vaccine effectiveness in the given country. As there is not yet consensus in the literature on a method to estimate the incidence of symptomatic COVID-19 cases, the same ratio between hospitalizations and cases is assumed for influenza and COVID-19. This is a crude assumption, but since it is uncertain whether COVID-19 has stabilized and it has not been possible to find post-pandemic case hospitalization rates (CHR) for the two diseases that are comparable, this is the simplest method for estimating COVID-19 incidence.

RSV incidence is calculated based on literature-based multiplication factors. Primary sector contacts are estimated with a ratio of 8.5 relative to hospital admissions among those aged ≥60 [45]. Episodes without medical contact are calculated based on a ratio of 2.27 between primary sector contacts and non-contact episodes [46,47].

PD is defined as cases of PD that are vaccine-preventable by the PCV23 vaccine. Based on the literature, the number of IPD cases and pneumococcal pneumonia cases have been estimated, along with the proportion of these cases caused by vaccine-preventable serotypes. Rates for Community Acquired Pneumonia (CAP) are taken from Birch et al. [18], and the proportion of these cases that are vaccine-preventable with the PCV23 vaccine is based on Lansbury et al. [48]. Likewise, the IPD rate is based on national surveillance, and the proportion of these cases that are vaccine-preventable are based on Statens Serum Institut [49,50].

All estimates for incidence consider the proportion of disease prevented through vaccination. Weighted vaccine effectiveness was calculated according to the distribution of vaccine products used within each national vaccination programmes, with product-specific vaccine effectiveness estimates weighted by their respective market shares. The resulting weighted vaccine effectiveness was subsequently combined with country-specific vaccine coverage to estimate the proportion of disease prevented through vaccination and thereby derive the underlying disease burden.

Hospital admissions

Data for Denmark for COVID-19, Influenza and RSV hospital admissions was derived from Statens Serum Institut from the 2024/2025 season, defined as laboratory confirmed admissions between week 21 2024 to week 20 2025 [51]. According to Bager et al. [28], COVID-19 testing was more frequent than influenza testing until the end of 2022, after which testing rates became more aligned. This is supported by the total number of PCR tests conducted in the 2024/2025 season: approximately 215,000 for COVID-19 and about 210,000 for influenza [51]. Therefore, no adjustment is made for differences in reporting between these two diseases, and raw estimates of laboratory-confirmed admissions are used without correction. Given that systematic testing has been implemented in Danish respiratory hospital settings, RSV testing in adults may still be incomplete in clinical practice, where testing can be omitted or yield lower diagnostic sensitivity. Therefore, adjustments were made using multiplication factors (MF) based on literature and expert opinion [32,51,52]. PD admissions were estimated as hospitalized IPD plus pneumococcal pneumonia. It was assumed that all IPD cases are admitted to the hospital. Pneumococcal pneumonia are estimated as the proportion of yearly CAP admissions in Denmark caused by vaccine-preventable serotypes [49,53,54]. For the other Nordic countries, hospital admissions were estimated using cross country extrapolation (see below).

Mortality

Data for Denmark for influenza and RSV mortality was derived from Statens Serum Institut from the 2024/2025 season, defined deaths within 30 days after laboratory confirmed influenza diagnosis from the Danish Civil Registration System (CPR register) between week 21 2024 to week 20 2025 [51]**.** The number of COVID-19 deaths was obtained from the European Respiratory Virus Surveillance Summary data for the 2024/2025 season [55]. 10% was added to the confirmed data to account for cases that were not tested per expert recommendation. PD mortality was estimated based on mortality rates identified in the literature for Pneumococcal pneumonia and IPD, and adjusted to be serotype specific [49,53,56,57]. For the other Nordic countries, mortality were estimated using cross country extrapolation (see below).

Cross-Country Extrapolation

The crude burden estimates for Denmark were extrapolated to Sweden, Norway, and Finland while taking literature as well as national statistics for each country on mortality and admissions into account. This was done by adjusting for key demographic and

immunological differences, including population size, vaccine uptake, and vaccine effectiveness (sources are available in table S 6). In cases where country specific values were lacking, an average of the remaining Nordic countries were used as proxies. By using Danish estimates as a reference, the analysis avoided the effects of heterogeneous surveillance intensity, test availability, and diagnostic culture across countries, ensuring that between-country differences were not driven by variations in the quality of the surveillance systems.

Economic estimates

Data Inputs and Sources

The calculations included direct medical costs, which comprised inpatient visits (overnight hospitalization admission), outpatient visits (hospital visit without overnight admission), and general practitioner (GP) consultations. Vaccination program costs, which included both the acquisition prices of vaccines and the administration costs. And lastly, indirect costs, assessed from a societal perspective, reflect productivity losses due to illness-related absenteeism.

All economic inputs were obtained from official national sources or peer-reviewed publications. Unit costs for healthcare services (GP consultations, outpatient visits and inpatient visits), were taken from cost-effectiveness literature, with most inputs being sourced from a multi-country study by Jacob et al. [2] comparing influenza vaccination strategies across Denmark, Sweden, and Norway. These unit costs and inputs were supplemented by a similar study by Alvares et al. [5] when cost input for Finland were missing. Average unit costs were assumed to be comparable across diseases to avoid introducing variation based on inconsistent methodologies in the health economic literature. Vaccine prices were sourced from official national price lists [58–62]. Productivity loss estimates were calculated using a human capital approach. In cases where country specific costs were lacking, estimates from the most economically similar country were used. All costs were converted to 2025 values. (All input sources are available in table S 6.3 and S 6.4)

S6 Input tables

Table S 6.1: Epidemiological inputs applied across all countries

Parameter	Value	Reference
**Influenza**		
Influenza attack rate in unvaccinated population	7.20%	[44]
Vaccine effectiveness		
SD-QIV VE against influenza A H1N1	62.00%	[2]
SD-QIV VE against influenza A H3N2	24.00%	[2]
SD-QIV VE against influenza B	63.00%	[2]
EIV VE against influenza A H1N1	79.00%	[2]
EIV VE against influenza A H3N2	31.00%	[2]
EIV VE against influenza B	81.00%	[2]
**COVID-19**		
Vaccine effectiveness	57.80%	[63]
**RSV**		
Vaccine effectiveness	87.50%	[64]
**PD**		
Vaccine serotype coverage		
PPV23 coverage of CAP isolates	13.30%	[53]
PPV23 coverage of IPD isolates	72.00%	[49]
Vaccine effectiveness		
PPV23 VE against CAP	18.00%	[65]
PPV23 VE against IPD	58.00%	[66]
PCV13 VE against CAP	45.00%	[65]
PCV13 VE against IPD	61.50%	[67]

EIV, enhanced influenza vaccine; CAP, community acquired pneumonia; IPD, invasive pneumococcal disease; PCV13, 13-valent pneumococcal conjugate vaccine; PD, pneumococcal disease; PPV23, 23-valent pneumococcal polysaccharide vaccine; RSV, respiratory syncytial virus; SD-QIV, standard-dose quadrivalent inactivated influenza vaccine; VE, vaccine effectiveness.

Table S 6.2: Country-specific epidemiological inputs.

Parameter	Denmark	Sweden	Norway	Finland	References
Population size (≥ 65 years)	1,250,183	2,311,090	1,060,363	1,328,817	DK: [68], SE: [69], NO: [70], FI: [71]
Vaccine coverage rate					
COVID-19	74.90%	72.80%	50.60%	49.70%	DK: [72], SE: [73], NO: [74], FI: [75]
Influenza	76.00%	69.00%	66.50%	61.50%	DK: [72], SE: [76], NO: [77], FI: [78]
RSV	0.70%	4.20%	4.20%	0.70%	DK: [79], FI, SE, and NO approximated from DK.
PD	1.10%	7.77%	10.00%	10.00%	DK: [79], FI: [80], NO and SE approximated from DK and FI.
Influenza strain distribution					
Influenza A (H1N1)	32.20%	32.20%	36.30%	34.60%	DK, NO, SV: [2] FI: average of DK, NO and SE
Influenza A (H3N2)	42.80%	41.30%	32.90%	39.00%	
Influenza B	25.00%	26.50%	30.70%	27.40%	

DK, Denmark; FI, Finland; NO, Norway; PD, pneumococcal disease; RSV, respiratory syncytial virus; SE, Sweden.

Table S 6.3: Economic inputs applied across all countries for estimating productivity costs.

Parameter	Value	Reference
Societal perspective inputs		
Number of days lost due to symptomatic VPRD	3.20	[2]
Number of days lost due to hospitalization	5.5	[81]

*All values are in days. See Table S6.4 below for country-specific economic inputs.

VRPD, vaccine preventable respiratory disease

Table S 6.4: Country-specific economic inputs.

Parameter	Denmark	Sweden	Norway	Finland	References
Vaccine costs					
SD-QIV	9.2	9.9	10.7	9.8	DK: [58], SE: [2], NO: [82], FI: [60]
EIV	26.6	26.6	26.6	26.6	Assumption based on prices from DK and NO
COVID-19	19.1	19.1	19.1	19.1	DK: [58], FI: [60], SE: FI, NO: DK
RSV	166.89	183.66	153.86	233.49	DK: [58], SE: [62], NO: [82], FI: [60]
PPV23	27.64	27.64	22.33	49.84	DK: [58] SE: [62], NO: [82], FI: [60]
PCV20	112.45	57.39	56.20	96.50	DK: [58], SE: [62], NO: [82] , FI: [60]
PCV13	-	-	44.16	-	NO: [82]
Vaccine admin costs	32.40	76.49	61.18	52.27	DK, SE, NO: [2]<br>FI: [5]
Hospitalization cost	4,876.84	7,272.67	63,701.99	4,257.29	DK, SE, NO: [2]<br>FI: [5]
Societal perspective inputs					
Proportion of the population employed (65–74 years)	16.60%	19.70%	19.20%	13.80%	DK, SE, NO: [2]<br>FI: [83]
Labor costs per day	328.71	330.68	279.82	319.00	DK, SE, NO: [2]<br>FI: SV

All costs and prices are in 2025 EUR

EIV, enhanced influenza vaccine (assumed to be average public purchase price of high-dose and adjuvanted vaccines); DK, Denmark; FI, Finland; NO, Norway; PCV13, 13-valent pneumococcal conjugate vaccine; PCV20, 20-valent pneumococcal conjugate vaccine; PPV23, 23-valent pneumococcal polysaccharide vaccine; RSV, respiratory syncytial virus; SD-QIV, standard-dose quadrivalent inactivated influenza vaccine; SE, Sweden.

S7 Overview of scenario analyses

Analysis	Assumption	Incidence, estimated crude (N)	Hospital admissions, estimated crude (N)	Mortality, estimated crude (N)	Total societal costs, €million
**Base case**	2024/2025 season	830,000	60,300	7,750	1,213
**Scenario 1**	2023/2024 season	1,207,000	67,200	10,270	1,264
**Scenario 2**	High influenza burden	1,023,000	74,500	9,320	1,336
**Scenario 3**	Low influenza burden	718,000	51,800	6,360	1,139
**Scenario 4**	Higher RSV burden	899,000	64,000	8,210	1,246
**Scenario 5**	COVID-19 vaccines at list price	830,000	60,300	7,750	1,475

S8 Epidemiological burden results

Scenario 1

Figure S 8.1: Bubble chart of the burden of COVID-19, Influenza, PD and RSV in terms of incidence, hospital admissions and mortality collectively in the Nordic Countries (Denmark, Sweden, Norway and Finland), in the 2023/2024 season.

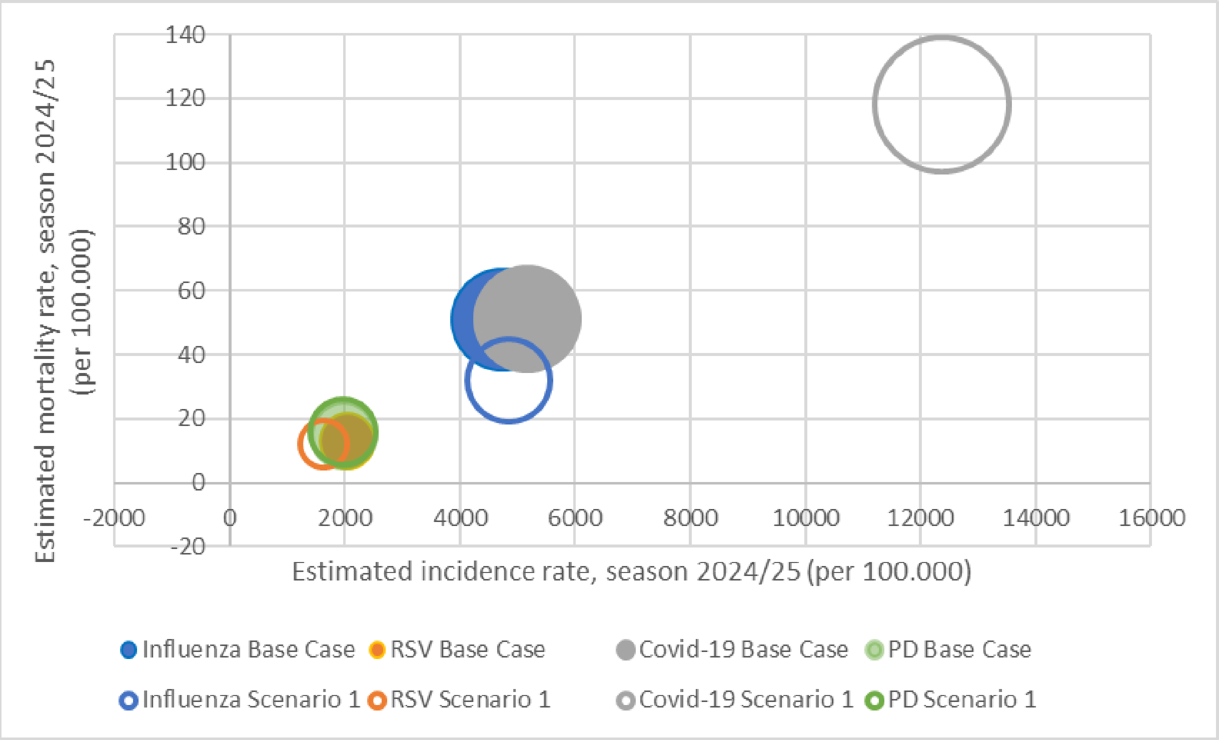

The diameter of the bubble reflects the number of hospital admissions per 100,000 people.

PD, pneumococcal disease; RSV, respiratory syncytial virus.

Table S 8.1: Epidemiological burden estimates of COVID-19, influenza, RSV and PD in the Nordic countries in season 2023/2024.

	Covid-19		Influenza		RSV		PD	
	**Estimated crude (N)**	**Estimated rate (per 100,000)**	**Estimated crude (N)**	**Estimated rate (per 100,000)**	**Estimated crude (N)**	**Estimated rate (per 100,000)**	**Estimated crude (N)**	**Estimated rate (per 100,000)**
**Denmark**								
Incidence	137,000	11,125	58,000	4,710	20,000	1,624	24,000	1,949
Admissions	7,200	585	3,000	244	1,100	89	2,100	171
Mortality	1,150	93	380	31	150	12	200	16
**Sweden**								
Incidence	252,000	11,421	108,000	4,895	36,000	1,632	44,000	1,994
Admissions	13,300	603	5,700	258	1,900	86	3,700	168
Mortality	2,890	131	710	32	270	12	360	16
**Norway**								
Incidence	143,000	13,801	50,000	4,825	17,000	1,641	20,000	1,930
Admissions	7,600	733	2,600	251	900	87	1,700	164
Mortality	1,210	117	330	32	130	13	160	15
**Finland**								
Incidence	183,000	13,976	67,000	5,117	22,000	1,680	26,000	1,986
Admissions	9,700	741	3,500	267	1,100	84	2,100	160
Mortality	1,550	118	430	33	160	12	190	15
**Total Nordics**								
Incidence	715,000	12,363	283,000	4,893	95,000	1,643	114,000	1,971
Admissions	37,800	654	14,800	256	5,000	86	9,600	166
Mortality	6,800	118	1,850	32	710	12	910	16

Incidence is rounded to the nearest 1,000, admissions are rounded to the nearest 100 and mortality is rounded to the nearest 10.

PD, pneumococcal disease; RSV, respiratory syncytial virus.

Scenario 2

Figure S 8.2: Bubble chart of the burden of COVID-19, Influenza, PD and RSV in terms of incidence, hospital admissions and mortality collectively in the Nordic Countries (Denmark, Sweden, Norway and Finland), assuming high influenza burden in the 2024/2025 season.

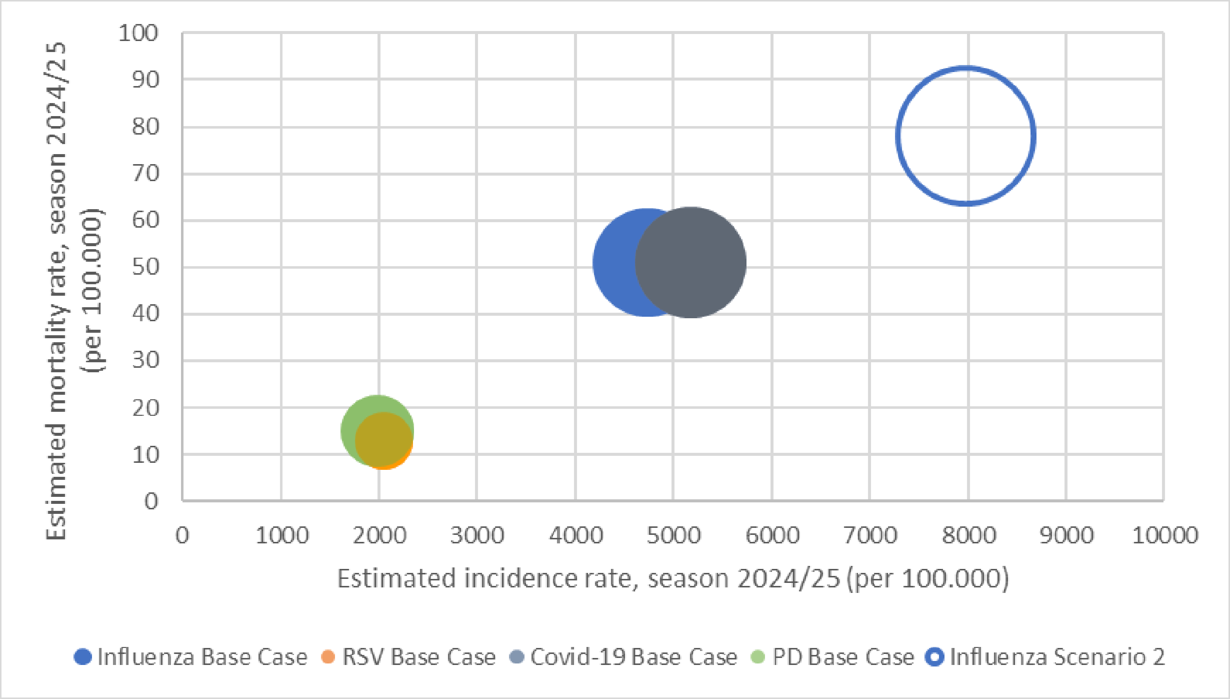

The diameter of the bubble reflects the number of hospital admissions per 100,000 people.

PD, pneumococcal disease; RSV, respiratory syncytial virus.

Table S 8.2: Epidemiological burden estimates of COVID-19, influenza, RSV and PD in the Nordic countries assuming high influenza burden in season 2024/2025.

	Covid-19		Influenza		RSV		PD	
	**Estimated crude (N)**	**Estimated rate (per 100,000)**	**Estimated crude (N)**	**Estimated rate (per 100,000)**	**Estimated crude (N)**	**Estimated rate (per 100,000)**	**Estimated crude (N)**	**Estimated rate (per 100,000)**
**Denmark**								
Incidence	58,000	4,639	87,000	6,959	26,000	2,080	25,000	2,000
Admissions	4,400	352	6,600	528	1,300	104	2,100	168
Mortality	470	38	860	69	160	13	200	16
**Sweden**								
Incidence	110,000	4,760	187,000	8,091	47,000	2,034	46,000	1,990
Admissions	8,300	359	14,100	610	2,400	104	3,800	164
Mortality	1,410	61	1,840	80	300	13	370	16
**Norway**								
Incidence	62,000	5,847	84,000	7,922	22,000	2,075	21,000	1,980
Admissions	4,600	434	6,300	594	1,100	104	1,700	160
Mortality	500	47	830	78	140	13	160	15
**Finland**								
Incidence	78,000	5,870	117,000	8,805	27,000	2,032	26,000	1,957
Admissions	5,900	444	8,400	632	1,400	105	2,100	158
Mortality	630	47	1,090	82	170	13	190	14
**Total Nordics**								
Incidence	308,000	5,176	475,000	7,983	122,000	2,050	118,000	1,983
Admissions	23,200	390	35,400	595	6,200	104	9,700	163
Mortality	3,010	51	4,620	78	770	13	920	15

Incidence is rounded to the nearest 1,000, admissions are rounded to the nearest 100 and mortality is rounded to the nearest 10.

PD, pneumococcal disease; RSV, respiratory syncytial virus.

Scenario 3

Figure S 8.3: Bubble chart of the burden of COVID-19, Influenza, PD and RSV in terms of incidence, hospital admissions and mortality collectively in the Nordic Countries (Denmark, Sweden, Norway and Finland), assuming low influenza burden in the 2024/2025 season.

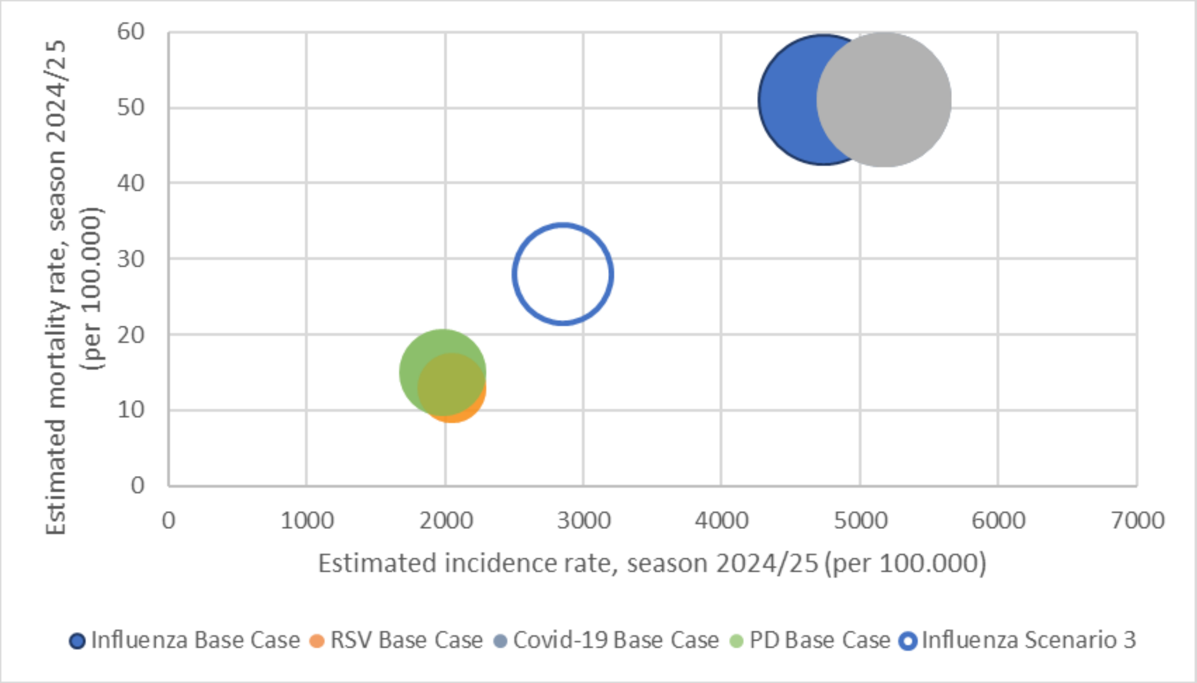

The diameter of the bubble reflects the number of hospital admissions per 100,000 people.

PD, pneumococcal disease; RSV, respiratory syncytial virus.

Table S 8.3: Epidemiological burden estimates of COVID-19, influenza, RSV and PD in the Nordic countries assuming low influenza burden in season 2024/2025

	Covid-19		Influenza		RSV		PD	
	**Estimated crude (N)**	**Estimated rate (per 100,000)**	**Estimated crude (N)**	**Estimated rate (per 100,000)**	**Estimated crude (N)**	**Estimated rate (per 100,000)**	**Estimated crude (N)**	**Estimated rate (per 100,000)**
**Denmark**								
Incidence	58,000	4,639	31,000	2,480	26,000	2,080	25,000	2,000
Admissions	4,400	352	2,400	192	1,300	104	2,100	168
Mortality	470	38	310	25	160	13	200	16
**Sweden**								
Incidence	110,000	4,760	67,000	2,899	47,000	2,034	46,000	1,990
Admissions	8,300	359	5,000	216	2,400	104	3,800	164
Mortality	1,410	61	660	29	300	13	370	16
**Norway**								
Incidence	62,000	5,847	30,000	2,829	22,000	2,075	21,000	1,980
Admissions	4,600	434	2,300	217	1,100	104	1,700	160
Mortality	500	47	300	28	140	13	160	15
**Finland**								
Incidence	78,000	5,870	42,000	3,161	27,000	2,032	26,000	1,957
Admissions	5,900	444	3,000	226	1,400	105	2,100	158
Mortality	630	47	390	29	170	13	190	14
**Total Nordics**								
Incidence	308,000	5,176	170,000	2,857	122,000	2,050	118,000	1,983
Admissions	23,200	390	12,700	213	6,200	104	9,700	163
Mortality	3,010	51	1,660	28	770	13	920	15

Incidence is rounded to the nearest 1,000, admissions are rounded to the nearest 100 and mortality is rounded to the nearest 10.

PD, pneumococcal disease; RSV, respiratory syncytial virus.

Scenario 4

Figure S 8.4: Bubble chart of the burden of COVID-19, Influenza, PD and RSV in terms of incidence, hospital admissions and mortality collectively in the Nordic Countries (Denmark, Sweden, Norway and Finland), assuming high under ascertainment of RSV in crude data in season 2024/2025.

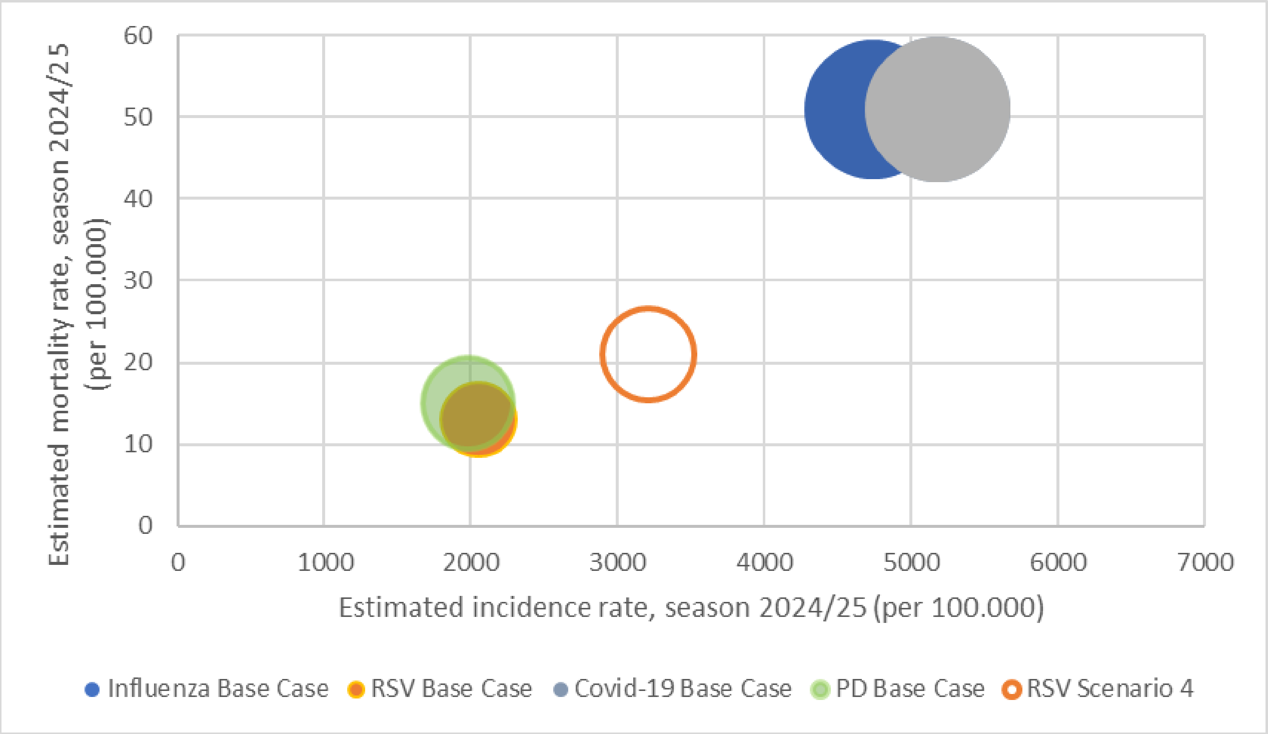

The diameter of the bubble reflects the number of hospital admissions per 100,000 people.

PD, pneumococcal disease; RSV, respiratory syncytial virus.

Table S 8.4: Epidemiological burden estimates of COVID-19, influenza, RSV and PD in the Nordic countries assuming higher RSV burden in season 2024/2025.

	Covid-19		Influenza		RSV		PD	
	Estimated crude (N)	Estimated rate (per 100,000)	Estimated crude (N)	Estimated rate (per 100,000)	Estimated crude (N)	Estimated rate (per 100,000)	Estimated crude (N)	Estimated rate (per 100,000)
**Denmark**								
Incidence	58,000	4,639	52,000	4,159	40,000	3,200	25,000	2,000
Admissions	4,400	352	3,900	312	2,100	168	2,100	168
Mortality	470	38	570	46	260	21	200	16
**Sweden**								
Incidence	110,000	4,760	112,000	4,846	74,000	3,202	46,000	1,990
Admissions	8,300	359	8,500	368	3,800	164	3,800	164
Mortality	1,410	61	1,210	52	480	21	370	16
**Norway**								
Incidence	62,000	5,847	51,000	4,810	34,000	3,206	21,000	1,980
Admissions	4,600	434	3,800	358	1,800	170	1,700	160
Mortality	500	47	550	52	220	21	160	15
**Finland**								
Incidence	78,000	5,870	67,000	5,042	43,000	3,236	26,000	1,957
Admissions	5,900	444	5,000	376	2,200	166	2,100	158
Mortality	630	47	720	54	270	20	190	14
**Total Nordics**								
Incidence	308,000	5,176	282,000	4,739	191,000	3,210	118,000	1,983
Admissions	23,200	390	21,200	356	9,900	166	9,700	163
Mortality	3,010	51	3,050	51	1,230	21	920	15

Incidence is rounded to the nearest 1,000, admissions are rounded to the nearest 100 and mortality is rounded to the nearest 10.

PD, pneumococcal disease; RSV, respiratory syncytial virus.

S9 Economic burden Results

Scenario 1

Figure S 9.1: Economic burden estimates of COVID-19, influenza, RSV and PD in the Nordic countries in season 2023/2024 in million euros.

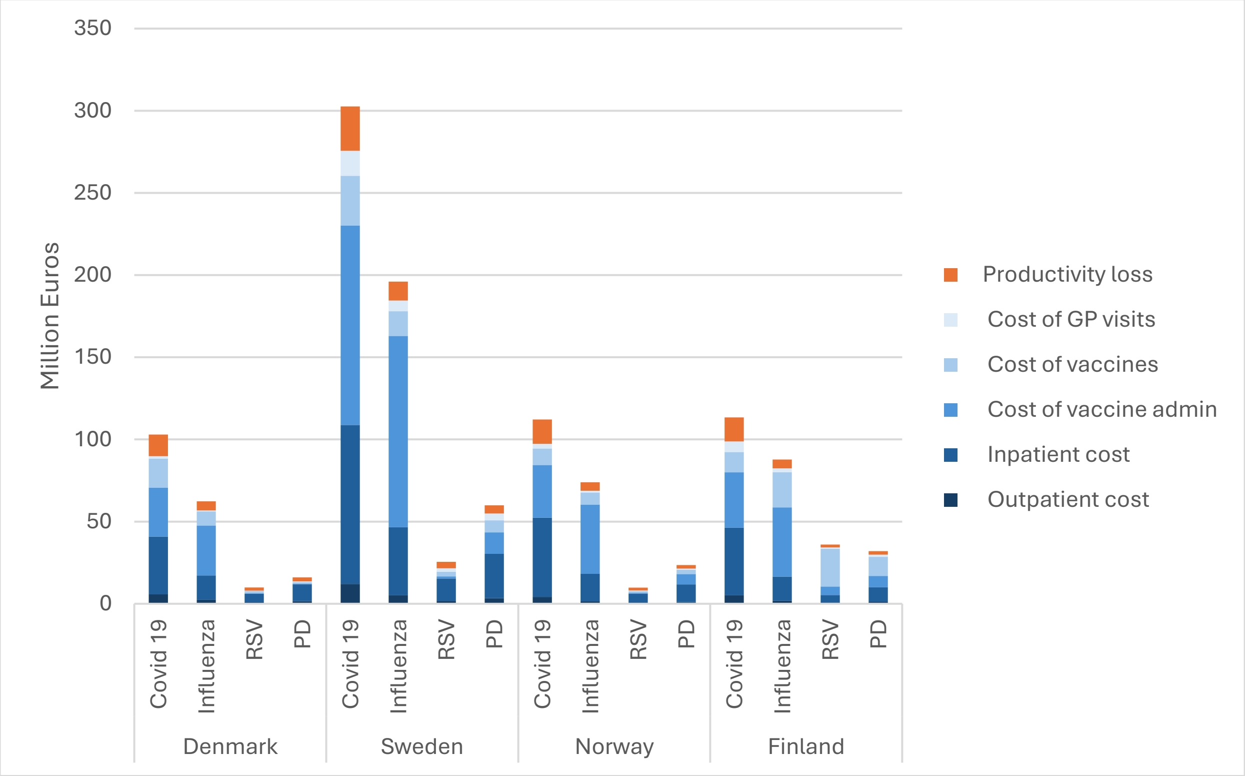

PD, pneumococcal disease; RSV, respiratory syncytial virus.

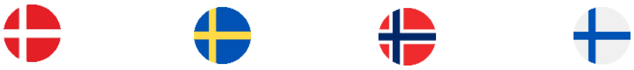

Table S 9.1: Economic burden estimates of COVID-19, influenza, RSV and PD in the Nordic countries season 2023/2024.

	Covid 19	Influenza	RSV	PD
**Denmark**				
Total Healthcare Costs	89,780,000	56,790,000	7,960,000	13,560,000
Cost of vaccines	17,620,000	8,620,000	1,470,000	920,000
Cost of vaccine admin	29,880,000	30,320,000	290,000	450,000
Cost of GP visits	1,420,000	600,000	210,000	410,000
Outpatient cost	5,710,000	2,410,000	840,000	1,650,000
Inpatient cost	35,150,000	14,840,000	5,160,000	10,130,000
Total Societal Costs	102,940,000	62,350,000	9,930,000	16,020,000
Productivity loss	13,160,000	5,560,000	1,960,000	2,460,000
**Sweden**				
Total Healthcare Costs	275,600,000	184,520,000	21,580,000	54,970,000
Cost of vaccines	30,280,000	15,070,000	2,840,000	7,290,000
Cost of vaccine admin	121,270,000	116,460,000	1,180,000	13,110,000
Cost of GP visits	15,200,000	6,490,000	2,150,000	4,230,000
Outpatient cost	12,030,000	5,140,000	1,700,000	3,350,000
Inpatient cost	96,820,000	41,360,000	13,700,000	26,980,000
Total Societal Costs	302,620,000	196,060,000	25,470,000	59,880,000
Productivity loss	27,030,000	11,540,000	3,890,000	4,900,000
**Norway**				
Total Healthcare Costs	97,280,000	68,710,000	8,020,000	21,370,000
Cost of vaccines	10,020,000	7,370,000	1,120,000	2,660,000
Cost of vaccine admin	32,080,000	42,130,000	440,000	6,340,000
Cost of GP visits	2,840,000	990,000	330,000	640,000
Outpatient cost	4,140,000	1,440,000	480,000	930,000
Inpatient cost	48,210,000	16,790,000	5,640,000	10,800,000
Total Societal Costs	112,110,000	73,870,000	9,780,000	23,580,000
Productivity loss	14,830,000	5,160,000	1,760,000	2,210,000
**Finland**				
Total Healthcare Costs	98,770,000	82,410,000	34,280,000	29,880,000
Cost of vaccines	12,340,000	21,430,000	23,030,000	11,560,000
Cost of vaccine admin	33,770,000	42,110,000	5,150,000	6,910,000
Cost of GP visits	6,450,000	2,310,000	750,000	1,400,000
Outpatient cost	5,110,000	1,830,000	590,000	1,110,000
Inpatient cost	41,100,000	14,730,000	4,760,000	8,910,000
Total Societal Costs	113,430,000	87,740,000	36,010,000	32,030,000
Productivity loss	14,660,000	5,340,000	1,730,000	2,140,000
**Nordic countries**				
Total Healthcare Costs	561,430,000	392,430,000	71,840,000	119,780,000
Cost of vaccines	70,260,000	52,490,000	28,460,000	22,430,000
Cost of vaccine admin	217,000,000	231,020,000	7,060,000	26,810,000

Cost of GP visits	25,910,000	10,390,000	3,440,000	6,680,000
Outpatient cost	26,990,000	10,820,000	3,610,000	7,040,000
Inpatient cost	221,280,000	87,720,000	29,260,000	56,820,000
Total Societal Costs	631,100,000	420,020,000	81,190,000	131,510,000
Productivity loss	69,680,000	27,600,000	9,340,000	11,710,000

All results are reported in euros as annual costs and rounded to nearest 10,000 euros.

GP, general practitioner; PD, pneumococcal disease; RSV, respiratory syncytial virus.

Scenario 2

Figure S 9.2: Economic burden estimates of COVID-19, influenza, RSV and PD in the Nordic countries assuming high influenza burden in season 2024/2025 in million euros.

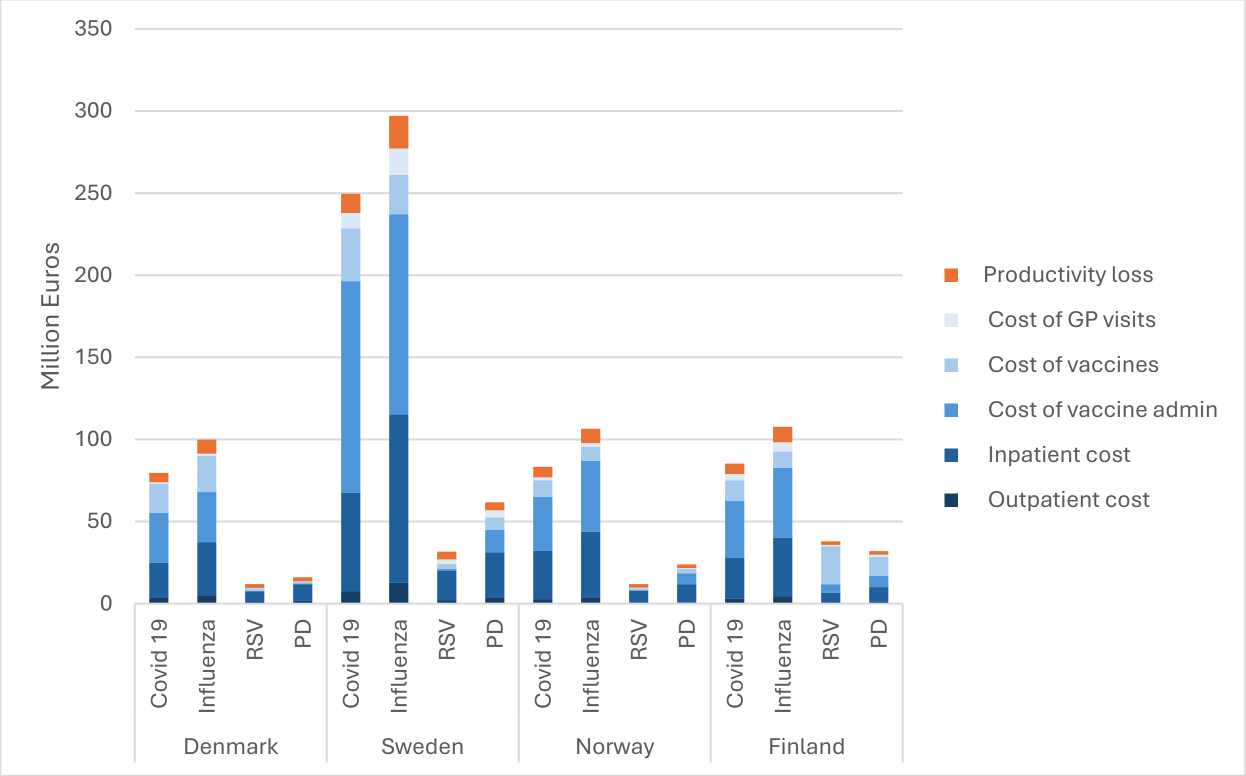

PD, pneumococcal disease; RSV, respiratory syncytial virus.

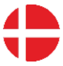
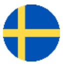
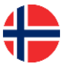
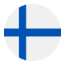

Table S 9.2: Economic burden estimates of COVID-19, influenza, RSV and PD in the Nordic countries assuming high influenza burden in season 2024/2025.

	Covid 19	Influenza	RSV	PD
**Denmark**				
**Total Healthcare Costs**	73,920,000	91,310,000	9,540,000	13,580,000
Cost of vaccines	17,880,000	22,000,000	1,470,000	920,000
Cost of vaccine admin	30,340,000	30,780,000	290,000	450,000
Cost of GP visits	870,000	1,300,000	260,000	410,000
Outpatient cost	3,470,000	5,200,000	1,050,000	1,650,000
Inpatient cost	21,370,000	32,020,000	6,470,000	10,150,000
Total Societal Costs	79,670,000	99,930,000	11,970,000	16,070,000
Productivity loss	5,750,000	8,620,000	2,430,000	2,490,000
**Sweden**				
Total Healthcare Costs	237,960,000	277,200,000	26,920,000	56,810,000
Cost of vaccines	32,160,000	23,990,000	2,970,000	7,640,000
Cost of vaccine admin	128,780,000	121,980,000	1,240,000	13,740,000
Cost of GP visits	9,430,000	16,080,000	2,780,000	4,340,000
Outpatient cost	7,470,000	12,730,000	2,200,000	3,440,000
Inpatient cost	60,110,000	102,430,000	17,730,000	27,660,000
Total Societal Costs	249,610,000	297,040,000	31,730,000	61,730,000
Productivity loss	11,650,000	19,840,000	4,800,000	4,920,000
**Norway**				
Total Healthcare Costs	76,890,000	97,770,000	9,760,000	21,700,000
Cost of vaccines	10,250,000	8,480,000	1,140,000	2,730,000
Cost of vaccine admin	32,830,000	43,110,000	450,000	6,490,000
Cost of GP visits	1,740,000	2,370,000	420,000	640,000
Outpatient cost	2,540,000	3,470,000	610,000	940,000
Inpatient cost	29,530,000	40,340,000	7,130,000	10,900,000
Total Societal Costs	83,350,000	106,580,000	11,930,000	23,920,000
Productivity loss	6,460,000	8,810,000	2,180,000	2,230,000
**Finland**				
Total Healthcare Costs	79,000,000	98,240,000	35,830,000	29,900,000
Cost of vaccines	12,600,000	9,870,000	23,030,000	11,560,000
Cost of vaccine admin	34,490,000	42,740,000	5,150,000	6,910,000
Cost of GP visits	3,910,000	5,590,000	940,000	1,400,000
Outpatient cost	3,100,000	4,430,000	740,000	1,110,000
Inpatient cost	24,910,000	35,610,000	5,970,000	8,920,000
Total Societal Costs	85,300,000	107,670,000	37,940,000	32,030,000
Productivity loss	6,300,000	9,430,000	2,110,000	2,140,000
**Nordic countries**				
Total Healthcare Costs	467,770,000	564,520,000	82,050,000	121,990,000
Cost of vaccines	72,890,000	64,340,000	28,610,000	22,850,000
Cost of vaccine admin	226,440,000	238,610,000	7,130,000	27,590,000

Cost of GP visits	15,950,000	25,340,000	4,400,000	6,790,000
Outpatient cost	16,580,000	25,830,000	4,600,000	7,140,000
Inpatient cost	135,920,000	210,400,000	37,300,000	57,630,000
Total Societal Costs	497,930,000	611,220,000	93,570,000	133,750,000
Productivity loss	30,160,000	46,700,000	11,520,000	11,780,000

All results are reported in euros as annual costs and rounded to nearest 10,000 euros.

GP, general practitioner; PD, pneumococcal disease; RSV, respiratory syncytial virus.

Scenario 3

Figure S 9.3: Economic burden estimates of COVID-19, influenza, RSV and PD in the Nordic countries assuming low influenza burden in season 2024/2025 in million euros.

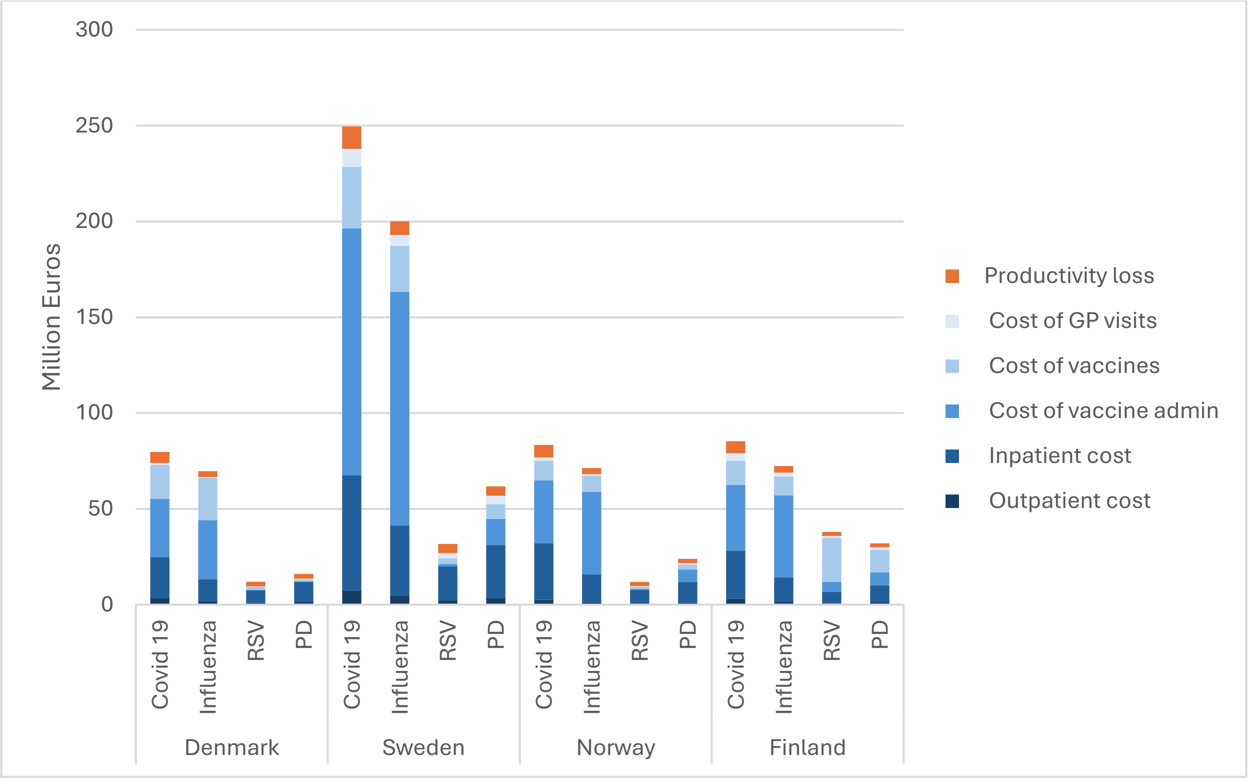

PD, pneumococcal disease; RSV, respiratory syncytial virus.

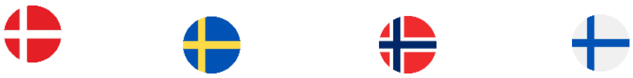

Table S 9.3: Economic burden estimates of COVID-19, influenza, RSV and PD in the Nordic countries assuming high influenza burden in season 2024/2025.

	Covid 19	Influenza	RSV	PD
**Denmark**				
**Total Healthcare Costs**	73,920,000	66,590,000	9,540,000	13,580,000
Cost of vaccines	17,880,000	22,000,000	1,470,000	920,000
Cost of vaccine admin	30,340,000	30,780,000	290,000	450,000
Cost of GP visits	870,000	460,000	260,000	410,000
**Outpatient cost**	3470000	1860000	1050000	1650000
Inpatient cost	21,370,000	11,480,000	6,470,000	10,150,000
Total Societal Costs	79,670,000	69,680,000	11,970,000	16,070,000
Productivity loss	5,750,000	3,090,000	2,430,000	2,490,000
**Sweden**				
Total Healthcare Costs	237,960,000	192,990,000	26,920,000	56,810,000
Cost of vaccines	32,160,000	23,990,000	2,970,000	7,640,000
Cost of vaccine admin	128,780,000	121,980,000	1,240,000	13,740,000
Cost of GP visits	9,430,000	5,760,000	2,780,000	4,340,000
Outpatient cost	7,470,000	4,560,000	2,200,000	3,440,000
Inpatient cost	60,110,000	36,700,000	17,730,000	27,660,000
Total Societal Costs	249,610,000	200,100,000	31,730,000	61,730,000
Productivity loss	11,650,000	7,110,000	4,800,000	4,920,000
**Norway**				
Total Healthcare Costs	76,890,000	68,140,000	9,760,000	21,700,000
Cost of vaccines	10,250,000	8,480,000	1,140,000	2,730,000
Cost of vaccine admin	32,830,000	43,110,000	450,000	6,490,000
Cost of GP visits	1,740,000	850,000	420,000	640,000
Outpatient cost	2,540,000	1,240,000	610,000	940,000
Inpatient cost	29,530,000	14,460,000	7,130,000	10,900,000
Total Societal Costs	83,350,000	71,300,000	11,930,000	23,920,000
Productivity loss	6,460,000	3,160,000	2,180,000	2,230,000
**Finland**				
Total Healthcare Costs	79,000,000	68,960,000	35,830,000	29,900,000
Cost of vaccines	12,600,000	9,870,000	23,030,000	11,560,000
Cost of vaccine admin	34,490,000	42,740,000	5,150,000	6,910,000
Cost of GP visits	3,910,000	2,000,000	940,000	1,400,000
Outpatient cost	3,100,000	1,590,000	740,000	1,110,000
Inpatient cost	24,910,000	12,760,000	5,970,000	8,920,000
Total Societal Costs	85,300,000	72,380,000	37,940,000	32,030,000
Productivity loss	6,300,000	3,410,000	2,110,000	2,140,000
**Nordic countries**				
Total Healthcare Costs	467,770,000	396,680,000	82,050,000	121,990,000
Cost of vaccines	72,890,000	64,340,000	28,610,000	22,850,000
Cost of vaccine admin	226,440,000	238,610,000	7,130,000	27,590,000

Cost of GP visits	15,950,000	9,070,000	4,400,000	6,790,000
Outpatient cost	16,580,000	9,250,000	4,600,000	7,140,000
Inpatient cost	135,920,000	75,400,000	37,300,000	57,630,000
Total Societal Costs	497,930,000	413,460,000	93,570,000	133,750,000
Productivity loss	30,160,000	16,770,000	11,520,000	11,780,000

All results are reported in euros as annual costs and rounded to nearest 10,000 euros.

GP, general practitioner; PD, pneumococcal disease; RSV, respiratory syncytial virus.

Scenario 4

Figure S. 9.4: Economic burden estimates of COVID-19, influenza, RSV and PD in the Nordic countries assuming high under ascertainment of RSV in crude data in season 2024/2025 in million euros.

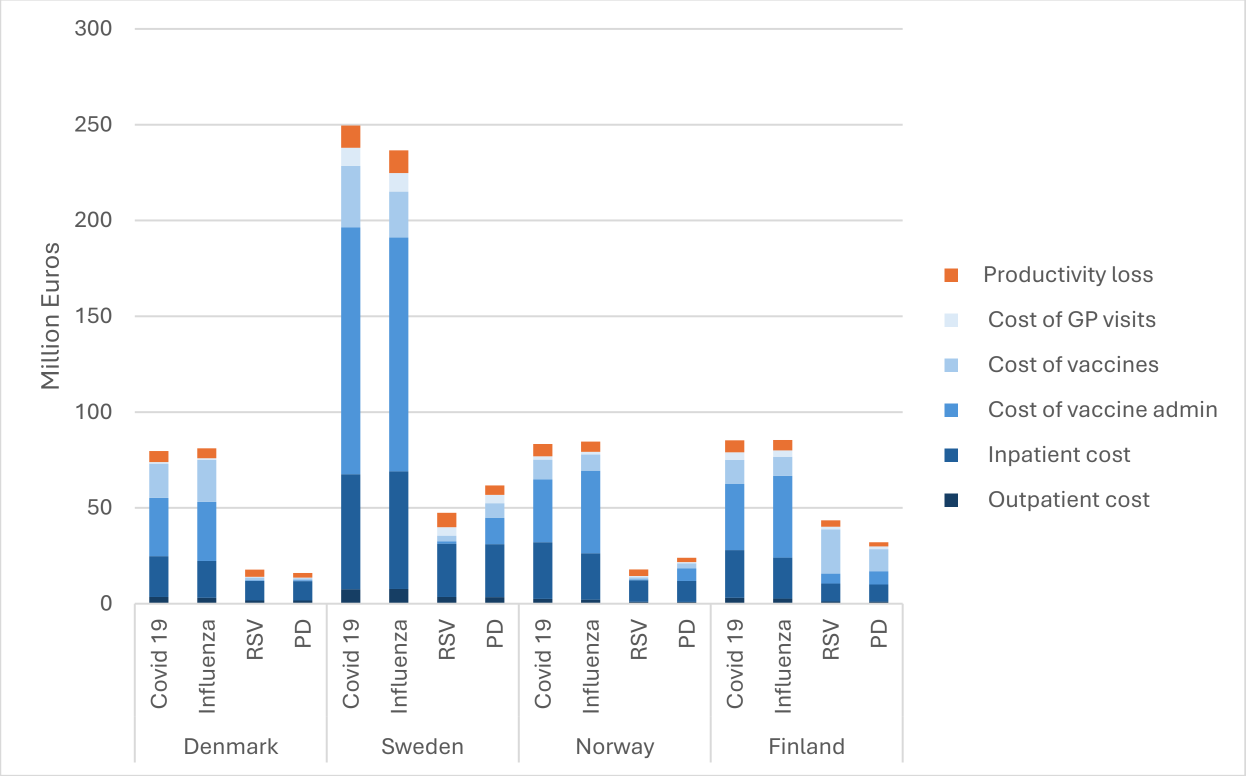

PD, pneumococcal disease; RSV, respiratory syncytial virus.

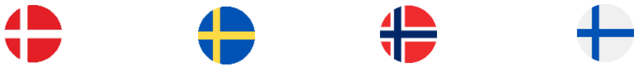

Table S 9.4: Economic burden estimates of COVID-19, influenza, RSV and PD in the Nordic countries assuming high influenza burden in season 2024/2025.

	Covid 19	Influenza	RSV	PD
**Denmark**				
**Total Healthcare Costs**	73,920,000	75,900,000	13,990,000	13,580,000
Cost of vaccines	17,880,000	22,000,000	1,470,000	920,000
Cost of vaccine admin	30,340,000	30,780,000	290,000	450,000
Cost of GP visits	870,000	780,000	410,000	410,000
**Outpatient cost**	3470000	3120000	1650000	1650000
Inpatient cost	21,370,000	19,210,000	10,170,000	10,150,000
Total Societal Costs	79,670,000	81,070,000	17,810,000	16,070,000
Productivity loss	5,750,000	5,170,000	3,830,000	2,490,000
**Sweden**				
Total Healthcare Costs	237,960,000	224,700,000	39,900,000	56,810,000
Cost of vaccines	32,160,000	23,990,000	2,970,000	7,640,000
Cost of vaccine admin	128,780,000	121,980,000	1,240,000	13,740,000
Cost of GP visits	9,430,000	9,650,000	4,370,000	4,340,000
Outpatient cost	7,470,000	7,640,000	3,460,000	3,440,000
Inpatient cost	60,110,000	61,460,000	27,860,000	27,660,000
Total Societal Costs	249,610,000	236,610,000	47,450,000	61,730,000
Productivity loss	11,650,000	11,910,000	7,550,000	4,920,000
**Norway**				
Total Healthcare Costs	76,890,000	79,300,000	14,420,000	21,700,000
Cost of vaccines	10,250,000	8,480,000	1,140,000	2,730,000
Cost of vaccine admin	32,830,000	43,110,000	450,000	6,490,000
Cost of GP visits	1,740,000	1,420,000	660,000	640,000
Outpatient cost	2,540,000	2,080,000	960,000	940,000
Inpatient cost	29,530,000	24,200,000	11,200,000	10,900,000
Total Societal Costs	83,350,000	84,580,000	17,840,000	23,920,000
Productivity loss	6,460,000	5,280,000	3,420,000	2,230,000
**Finland**				
Total Healthcare Costs	79,000,000	79,990,000	40,200,000	29,900,000
Cost of vaccines	12,600,000	9,870,000	23,030,000	11,560,000
Cost of vaccine admin	34,490,000	42,740,000	5,150,000	6,910,000
Cost of GP visits	3,910,000	3,350,000	1,470,000	1,400,000
Outpatient cost	3,100,000	2,660,000	1,170,000	1,110,000
Inpatient cost	24,910,000	21,370,000	9,380,000	8,920,000
Total Societal Costs	85,300,000	85,390,000	43,510,000	32,030,000
Productivity loss	6,300,000	5,410,000	3,320,000	2,140,000
**Nordic countries**				
Total Healthcare Costs	467,770,000	459,890,000	108,510,000	121,990,000
Cost of vaccines	72,890,000	64,340,000	28,610,000	22,850,000
Cost of vaccine admin	226,440,000	238,610,000	7,130,000	27,590,000

Cost of GP visits	15,950,000	15,200,000	6,910,000	6,790,000
Outpatient cost	16,580,000	15,500,000	7,240,000	7,140,000
Inpatient cost	135,920,000	126,240,000	58,610,000	57,630,000
Total Societal Costs	497,930,000	487,650,000	126,610,000	133,750,000
Productivity loss	30,160,000	27,770,000	18,120,000	11,780,000

All results are reported in euros as annual costs and rounded to the nearest 10,000 euros.

GP, general practitioner; PD, pneumococcal disease; RSV, respiratory syncytial virus.

Scenario 5

Figure S. 9.5: Economic burden estimates of COVID-19, influenza, RSV and PD in the Nordic countries assuming no discount of COVID-19 list prices in season 2024/2025 in million euros.

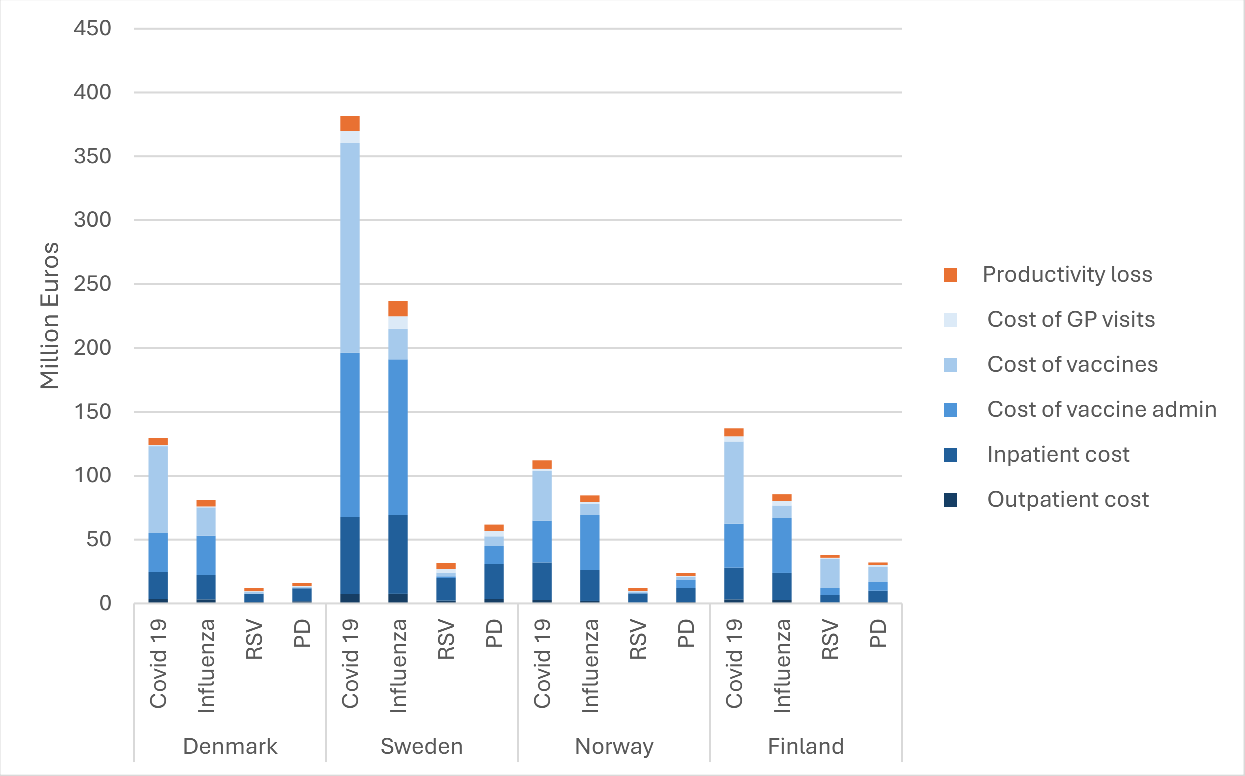

PD, pneumococcal disease; RSV, respiratory syncytial virus.

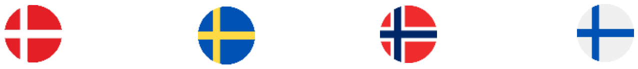

Table S 9.5: Economic burden estimates of COVID-19, influenza, RSV and PD in the Nordic countries assuming no discount on COVID-19 list prices in season 2024/2025.

	Covid 19	Influenza	RSV	PD
**Denmark**				
**Total Healthcare Costs**	123,920,000	75,900,000	9,540,000	13,580,000
Cost of vaccines	67,890,000	22,000,000	1,470,000	920,000
Cost of vaccine admin	30,340,000	30,780,000	290,000	450,000
Cost of GP visits	870,000	780,000	260,000	410,000
Outpatient cost	3,470,000	3,120,000	1,050,000	1,650,000
Inpatient cost	21,370,000	19,210,000	6,470,000	10,150,000
Total Societal Costs	129,670,000	81,070,000	11,970,000	16,070,000
Productivity loss	5,750,000	5,170,000	2,430,000	2,490,000
**Sweden**				
Total Healthcare Costs	369,910,000	224,700,000	26,920,000	56,810,000
Cost of vaccines	164,110,000	23,990,000	2,970,000	7,640,000
Cost of vaccine admin	128,780,000	121,980,000	1,240,000	13,740,000
Cost of GP visits	9,430,000	9,650,000	2,780,000	4,340,000
Outpatient cost	7,470,000	7,640,000	2,200,000	3,440,000
Inpatient cost	60,110,000	61,460,000	17,730,000	27,660,000
Total Societal Costs	381,570,000	236,610,000	31,730,000	61,730,000
Productivity loss	11,650,000	11,910,000	4,800,000	4,920,000
**Norway**				
Total Healthcare Costs	105,550,000	79,300,000	9,760,000	21,700,000
Cost of vaccines	38,900,000	8,480,000	1,140,000	2,730,000
Cost of vaccine admin	32,830,000	43,110,000	450,000	6,490,000
Cost of GP visits	1,740,000	1,420,000	420,000	640,000
Outpatient cost	2,540,000	2,080,000	610,000	940,000
Inpatient cost	29,530,000	24,200,000	7,130,000	10,900,000
Total Societal Costs	112,000,000	84,580,000	11,930,000	23,920,000
Productivity loss	6,460,000	5,280,000	2,180,000	2,230,000
**Finland**				
Total Healthcare Costs	130,710,000	79,990,000	35,830,000	29,900,000
Cost of vaccines	64,320,000	9,870,000	23,030,000	11,560,000
Cost of vaccine admin	34,490,000	42,740,000	5,150,000	6,910,000
Cost of GP visits	3,910,000	3,350,000	940,000	1,400,000
Outpatient cost	3,100,000	2,660,000	740,000	1,110,000
Inpatient cost	24,910,000	21,370,000	5,970,000	8,920,000
Total Societal Costs	137,020,000	85,390,000	37,940,000	32,030,000
Productivity loss	6,300,000	5,410,000	2,110,000	2,140,000
**Nordic countries**				
Total Healthcare Costs	730,090,000	459,890,000	82,050,000	121,990,000
Cost of vaccines	335,220,000	64,340,000	28,610,000	22,850,000
Cost of vaccine admin	226,440,000	238,610,000	7,130,000	27,590,000

Cost of GP visits	15,950,000	15,200,000	4,400,000	6,790,000
Outpatient cost	16,580,000	15,500,000	4,600,000	7,140,000
Inpatient cost	135,920,000	126,240,000	37,300,000	57,630,000
Total Societal Costs	760,260,000	487,650,000	93,570,000	133,750,000
Productivity loss	30,160,000	27,770,000	11,520,000	11,780,000

All results are reported in euros as annual costs and rounded to the nearest 10,000 euros.

GP, general practitioner; PD, pneumococcal disease; RSV, respiratory syncytial virus.

S10 References (supplementary)

[1] Poukka E, Perälä J, Nohynek H, et al. Relative effectiveness of bivalent boosters against severe COVID-19 outcomes among people aged ≥ 65 years in Finland, September 2022 to August 2023. Eurosurveillance [Internet]. 2024 [cited 2025 May 1];29(37).

[2] Jacob J, Biering-Sørensen T, Holger Ehlers L, et al. Cost-Effectiveness of Vaccination of Older Adults with an MF59®-Adjuvanted Quadrivalent Influenza Vaccine Compared to Standard-Dose and High-Dose Vaccines in Denmark, Norway, and Sweden. Vaccines. 2023;11(4):753.

[3] Hauge SH, Bakken IJ, De Blasio BF, et al. Burden of medically attended influenza in Norway 2008-2017. Influenza Other Respir Viruses. 2019;13(3):240–247.

[4] Emborg H-D, Bolt Botnen A, Nielsen J, et al. Age-dependent influenza infection patterns and subtype circulation in Denmark, in seasons 2015/16 to 2021/22. Eurosurveillance [Internet]. 2024 [cited 2025 Apr 28];29(4).

[5] Alvarez FP, Chevalier P, Borms M, et al. Cost-effectiveness of influenza vaccination with a high dose quadrivalent vaccine of the elderly population in Belgium, Finland, and Portugal. J Med Econ. 2023;26(1):710–719.

[6] Baum U, Kulathinal S, Auranen K. Spotlight influenza: Estimation of influenza vaccine effectiveness in elderly people with assessment of residual confounding by negative control outcomes, Finland, 2012/13 to 2019/20. Eurosurveillance [Internet]. 2021 [cited 2025 Apr 28];26(36).

[7] Li X, Willem L, Johannesen CK, et al. Influential drivers of the cost-effectiveness of respiratory syncytial virus vaccination in European older adults: a multi-country analysis. BMC Med. 2025;23(1):170.

[8] Rehnberg J, Glantz H, Telerud Vaerlien L. Abrysvo (RSV prefusion-F-pro-tein, subgrupp A och B) [Internet]. Stockholm: Tandvårds- och läkemedelsförmånsverket (TLV); 2023 [cited 2025 Apr 30]. Available from: www.tlv.se.

[9] Rehnberg J, Lindström L, Telerud Vaerlien L. Arexvy (RSV prefusion-F-protein, rekombinant) [Internet]. Stockholm: Tandvårds- och läkemedelsförmånsverket (TLV); 2023 [cited 2025 Apr 30]. Available from: www.tlv.se.

[10] Malene B M, Oyvind H, Tor M, et al. Cost-effectiveness of 20-valent pneumococcal conjugate vaccine compared with 23-valent pneumococcal polysaccharide vaccine among adults in a Norwegian setting. Cost Eff Resour Alloc. 2023;21(1):52.

[11] Steens A, Eriksen H-M, Blystad H. What are the most important infectious diseases among those ≥65 years: a comprehensive analysis on notifiable diseases, Norway, 1993–2011. BMC Infect Dis. 2014;14(1):57.

[12] Lyngstad TM, Kristoffersen AB, Winje BA, et al. Estimation of the incidence of hospitalisation for non-invasive pneumococcal pneumonia in the Norwegian population aged 50 years and older. Epidemiol Infect. 2022;150:e81.

[13] Nymark LS, Dag Berild J, Lyngstad TM, et al. Cost-utility analysis of a universal pneumococcal vaccination programme for older adults in Norway. Hum Vaccines Immunother. 2022;18(6):2101333.

[14] Palmborg A, Skovdal M, Molden T, et al. Invasive pneumococcal disease among the elderly in the later era of paediatric pneumococcal conjugate vaccination—A longitudinal study over 10 years based on public surveillance data in the Nordics. Melo-Cristino J, editor. PLOS ONE. 2023;18(6):e0287378.

[15] Winje BA, Vestrheim DF, White RA, et al. The Risk of Invasive Pneumococcal Disease Differs between Risk Groups in Norway Following Widespread Use of the 13-Valent Pneumococcal Vaccine in Children. Microorganisms. 2021;9(8):1774.

[16] Sevilla JP, Stawasz A, Burnes D, et al. Indirect costs of adult pneumococcal disease and productivity-based rate of return to PCV13 vaccination for older adults and elderly diabetics in Denmark. J Econ Ageing. 2019;14:100203.

[17] Olsen J, Schnack H, Skovdal M, et al. Cost-effectiveness of 20-valent pneumococcal conjugate vaccine in Denmark compared with PPV23. J Med Econ. 2022;25(1):1240–1254.

[18] Birck AM, Nordin Christensen L, Pedersen MH, et al. Health economic evaluation of introducing a PPSV23-based vaccination programme to adults aged 65 and above, and an extension to the 60-64 age group in Denmark. Expert Rev Vaccines. 2021;20(10):1327–1337.

[19] Wolff E, Storsaeter J, Örtqvist Å, et al. Cost-effectiveness of pneumococcal vaccination for elderly in Sweden. Vaccine. 2020;38(32):4988–4995.

[20] Zarabi N, Aldvén M, Sjölander S, et al. Clinical and economic burden of pneumococcal disease among adults in Sweden: A population-based register study. Shaw T, editor. PLOS ONE. 2023;18(7):e0287581.

[21] Naucler P, Henriques-Normark B, Hedlund J, et al. The changing epidemiology of community-acquired pneumonia: nationwide register-based study in Sweden. J Intern Med. 2019;286(6):689–701.

[22] Naucler P, Galanis I, Morfeldt E, et al. Comparison of the Impact of Pneumococcal Conjugate Vaccine 10 or Pneumococcal Conjugate Vaccine 13 on Invasive Pneumococcal Disease in Equivalent Populations. Clin Infect Dis. 2017;65(11):1780-1790.e1.

[23] Backhaus E, Berg S, Andersson R, et al. Epidemiology of invasive pneumococcal infections: manifestations, incidence and case fatality rate correlated to age, gender and risk factors. BMC Infect Dis. 2016;16(1):367.

[24] Pugh S, Wasserman M, Moffatt M, et al. Estimating the Impact of Switching from a Lower to Higher Valent Pneumococcal Conjugate Vaccine in Colombia, Finland, and The Netherlands: A Cost-Effectiveness Analysis. Infect Dis Ther. 2020;9(2):305–324.

[25] Polkowska A, Rinta-Kokko H, Toropainen M, et al. Long-term population effects of infant 10-valent pneumococcal conjugate vaccination on pneumococcal meningitis in Finland. Vaccine. 2021;39(23):3216–3224.

[26] Nuorti JP, Rinta-Kokko H, Toropainen M, et al. Long-term population impact of infant 10-valent pneumococcal conjugate vaccination on invasive pneumococcal disease in adults in Finland. Vaccine. 2022;40(41):5950–5958.

[27] Seppälä E, Bøås H, Dahl J, et al. Registry-Based Surveillance of Severe Acute Respiratory Infections in Norway During 2021–2024. Influenza Other Respir Viruses. 2025;19(2):e70080.

[28] Bager P, Svalgaard IB, Lomholt FK, et al. The hospital and mortality burden of COVID-19 compared with influenza in Denmark: a national observational cohort study, 2022–24. Lancet Infect Dis. 2025;S1473309924008065.

[29] Nielsen J, Krause TG, Mølbak K. Influenza-associated mortality determined from all-cause mortality, Denmark 2010/11-2016/17: The FluMOMO model. Influenza Other Respir Viruses. 2018;12(5):591–604.

[30] Nersesjan V, Amiri M, Christensen HK, et al. Thirty-Day Mortality and Morbidity in COVID-19 Positive vs. COVID-19 Negative Individuals and vs. Individuals Tested for Influenza A/B: A Population-Based Study. Front Med. 2020;7:598272.

[31] SST. Medicinsk teknologivurdering (MTV) af influenzavaccination [Internet]. København: Sundhedsstyrelsen (SST); 2021. Report No.: Version 2. Available from: ISBN: 978-87-7014-374-5.

[32] Urchueguía-Fornes A, Muñoz-Quiles C, Mira-Iglesias A, et al. Ten-Year Surveillance of Respiratory Syncytial Virus Hospitalizations in Adults: Incidence Rates and Case Definition Implications. J Infect Dis. 2025;231(5):e830–e839.

[33] López-Lacort M, Amini M, Emborg H-D, et al. Incidence of Invasive and Noninvasive Pneumococcal Pneumonia Hospitalizations in People Aged ≥50 Years: Assessing Variability Across Denmark and Spain. J Infect Dis. 2024;230(3):e559–e567.

[34] Seppälä E, Dahl J, Veneti L, et al. Covid-19 and influenza vaccine effectiveness against associated hospital admission and death among individuals over 65 years in Norway: A population-based cohort study, 3 October 2022 to 20 June 2023. Vaccine. 2024;42(3):620–628.

[35] Debes S, Haug JB, De Blasio BF, et al. Etiology of viral respiratory tract infections in hospitalized adults, and evidence of the high frequency of prehospitalization antibiotic treatment in Norway. Health Sci Rep. 2021;4(4):e403.

[36] Auvinen R, Syrjänen R, Ollgren J, et al. Clinical characteristics and population-based attack rates of respiratory syncytial virus versus influenza hospitalizations among adults—An observational study. Influenza Other Respir Viruses. 2022;16(2):276–288.

[37] Halme J, Syrjänen RK, Baum U, et al. Effectiveness of trivalent influenza vaccines against hospitalizations due to laboratory-confirmed influenza a in the elderly: Comparison of test-negative design with register-based designs. Vaccine. 2022;40(31):4242–4252.

[38] Osei-Yeboah R, Johannesen CK, Egeskov-Cavling AM, et al. Respiratory Syncytial Virus–Associated Hospitalization in Adults With Comorbidities in 2 European Countries: A Modeling Study. J Infect Dis. 2024;229(Supplement_1):S70–S77.

[39] Osei-Yeboah R, Spreeuwenberg P, Del Riccio M, et al. Estimation of the Number of Respiratory Syncytial Virus–Associated Hospitalizations in Adults in the European Union. J Infect Dis. 2023;228(11):1539–1548.

[40] Johannesen CK, Van Wijhe M, Tong S, et al. Age-Specific Estimates of Respiratory Syncytial Virus-Associated Hospitalizations in 6 European Countries: A Time Series Analysis. J Infect Dis. 2022;226(Supplement_1):S29–S37.

[41] Johannesen CK, Gideonse D, Osei-Yeboah R, et al. Estimation of respiratory syncytial virus-associated hospital admissions in five European countries: a modelling study. Lancet Reg Health - Eur. 2025;51:101227.

[42] Urchueguía-Fornes A, Osei-Yeboah R, Jollivet O, et al. RSV healthcare burden in adults before and since the emergence of the COVID-19 pandemic in 6 European

countries [Internet]. 2024 [cited 2025 May 4]. Available from: http://medrxiv.org/lookup/doi/10.1101/2024.09.20.24314093.

[43] de Fougerolles TR, Baïssas T, Perquier G, et al. Public health and economic benefits of seasonal influenza vaccination in risk groups in France, Italy, Spain and the UK: state of play and perspectives. BMC Public Health. 2024;24(1):1222.

[44] Somes MP, Turner RM, Dwyer LJ, et al. Estimating the annual attack rate of seasonal influenza among unvaccinated individuals: A systematic review and meta-analysis. Vaccine. 2018;36(23):3199–3207.

[45] Savic M, Penders Y, Shi T, et al. Respiratory syncytial virus disease burden in adults aged 60 years and older in high-income countries: A systematic literature review and meta-analysis. Influenza Other Respir Viruses. 2023;17(1):e13031.

[46] Korsten K, Adriaenssens N, Coenen S, et al. Burden of respiratory syncytial virus infection in community-dwelling older adults in Europe (RESCEU): an international prospective cohort study. Eur Respir J. 2021;57(4):2002688.

[47] Mao Z, Li X, Korsten K, et al. Economic Burden and Health-Related Quality of Life of Respiratory Syncytial Virus and Influenza Infection in European Community-Dwelling Older Adults. J Infect Dis. 2022;226(Supplement_1):S87–S94.

[48] Lansbury L, Lim B, McKeever TM, et al. Non-invasive pneumococcal pneumonia due to vaccine serotypes: A systematic review and meta-analysis. EClinicalMedicine. 2022;44:101271.

[49] Statens Serum Institut. Invasiv pneumokoksygdom - opgørelse over sygdomsforekomst 2020-23 [Internet]. ssi.dk. 2024 [cited 2025 Jul 15]. Available from: https://www.ssi.dk/sygdomme-beredskab-og-forskning/sygdomsovervaagning/i/invasiv-pneumokoksygdom---opgorelse-over-sygdomsforekomst-2020-23.

[50] Pneumokoksygdom [Internet]. www.ssi.dk. 2025 [cited 2025 Jul 15]. Available from: https://www.ssi.dk/vaccinationer/risikogrupper/pneumokoksygdom.

[51] Statens Serum Institut. Overvågning af influenza, covid-19, RS-virus og andre luftvejssygdomme [Internet]. ssi.dk. 2025 [cited 2025 Jul 15]. Available from: https://www.ssi.dk/sygdomme-beredskab-og-forskning/overvaagning-af-luftvejssygdomme.

[52] Statens Serum Institut. RS-virus [Internet]. experience.arcgis.com. [cited 2025 May 1]. Available from:

https://experience.arcgis.com/experience/220fef27d07d438889d651cc2e00076c/page/RS-virus/.

[53] Bahrs C, Kesselmeier M, Kolditz M, et al. A longitudinal analysis of pneumococcal vaccine serotypes in pneumonia patients in Germany. Eur Respir J. 2022;59(2):2102432.

[54] Danmarks statistik. Statistikbanken [Internet]. DST.dk. [cited 2025 Jun 15]. Available from: https://www.statistikbanken.dk/statbank5a/default.asp?w=1920.

[55] ERVISS, European Respiratory Virus Surveillance Summary. Country summary [Internet]. erviss.org. [cited 2025 Jul 1]. Available from: https://erviss.org/.

[56] Graversen SB, Pedersen HS, Sandbaek A, et al. Factors Associated With 30-Day Rehospitalization and Mortality in Older Patients After a Pneumonia Admission. J Am Med Dir Assoc. 2020;21(12):1869-1878.e10.

[57] Chen H, Matsumoto H, Horita N, et al. Prognostic factors for mortality in invasive pneumococcal disease in adult: a system review and meta-analysis. Sci Rep [Internet]. 2021 [cited 2025 Jul 16];11(1).

[58] Lægemiddelstyrelsen. Medicinpriser.dk [Internet]. 2025 [cited 2025 Feb 4]. Available from: https://www.medicinpriser.dk/.

[59] Pfizer, Moderna raise prices for its COVID-19 vaccines in EU [Internet]. Reuters. [cited 2026 Mar 31]. Available from: https://www.reuters.com/business/healthcare-pharmaceuticals/pfizer-moderna-raises-prices-its-covid-19-vaccines-eu-ft-2021-08-01/.

[60] Kansaneläkelaitos - The Social Insurance Institution of Finland. Medicinal Products Database [Internet]. asiointi.kela.fi. [cited 2025 Jun 4]. Available from: https://asiointi.kela.fi/laakekys_app/LaakekysApplication?kieli=en.

[61] Norwegian Medical Products Agency. Maximum price [Internet]. 2025 [cited 2026 Feb 16]. Available from: https://www.dmp.no/en/public-funding-and-pricing/pricing-of-medicines/maximum-price.

[62] Apoteket AB. Apoteket [Internet]. www.apoteket.se. [cited 2025 Jun 4]. Available from: https://www.apoteket.se/kategori/.

[63] Anderssen H. Prioriteringsmeldingen: – Nødvendig at vi prioriterer helsehjelp til pasienter i arbeidsfør alder [Internet]. 2024 [cited 2025 Feb 6]. Available from: https://www.healthtalk.no/healthtalk/prioriteringsmeldingen-nodvendig-at-vi-prioriterer-helsehjelp-til-pasienter-i-arbeidsfor-alder/182265.

[64] Papi A, Ison MG, Langley JM, et al. Respiratory Syncytial Virus Prefusion F Protein Vaccine in Older Adults. N Engl J Med. 2023;388(7):595–608.

[65] Farrar JL, Childs L, Ouattara M, et al. Systematic Review and Meta-Analysis of the Efficacy and Effectiveness of Pneumococcal Vaccines in Adults [Internet]. Cold Spring Harbor Laboratory; 2022 [cited 2025 Jul 16]. Available from: http://medrxiv.org/lookup/doi/10.1101/2022.10.06.22280772.

[66] Nielsen KF, Nielsen LB, Lomholt FK, et al. Effectiveness of the 23-valent pneumococcal polysaccharide vaccine against invasive pneumococcal disease among 948,263 individuals ≥65 years of age: A Danish cohort study [Internet]. Springer Science and Business Media LLC; 2022 [cited 2025 Jul 16]. Available from: https://www.researchsquare.com/article/rs-1715668/v1.

[67] Hsiao A, Lewis N, Hansen J, et al. Effectiveness of 13-valent pneumococcal conjugate vaccine against vaccine-type invasive pneumococcal disease in older adults. Vaccine. 2025;44:126543.

[68] Danmarks Statistik. Statistikbanken [Internet]. statistikbanken.dk. [cited 2025 May 1]. Available from: https://www.statistikbanken.dk/statbank5a/selectvarval/define.asp?PLanguage=0&subword=tabsel&MainTable=FOLK1AM&PXSId=239851&tablestyle=&ST=SD&buttons=0.

[69] Statistisk Centralbanken. Statistikdatabase [Internet]. statistikdatabasen.scb.se. [cited 2025 May 1]. Available from: https://www.statistikdatabasen.scb.se/pxweb/sv/ssd/START__BE__BE0101__BE0101A/BefolkManadCKM/.

[70] Statistics Norway. Statbank Norway [Internet]. ssb.no. [cited 2025 May 1]. Available from: https://www.ssb.no/en/statbank/table/07459/.

[71] Tilastokeskus. Tilastokeskus [Internet]. stat.fi. [cited 2025 May 1]. Available from: https://pxdata.stat.fi/PxWeb/pxweb/fi/.

[72] Statens Serum Institut. Influenza - Høj tilslutning og ny vaccine har beskyttet de sårbare [Internet]. ssi.dk. 2025 [cited 2025 May 1]. Available from: https://www.ssi.dk/aktuelt/nyheder/2025/hoj-tilslutning-og-ny-vaccine-har-beskyttet-de-saarbare.

[73] European Centre for Disease Prevention and Control. COVID-19 vaccination coverage in the EU/EEA during the 2023–24 season campaigns 1 September 2023 to

31 July 2024. [Internet]. LU: Publications Office; 2024 [cited 2025 Jul 16]. Available from: https://data.europa.eu/doi/10.2900/1677733.

[74] Folkehelseinstituttet (FHI). Statistikk [Internet]. statistikk.fhi.no. [cited 2025 May 1]. Available from: https://statistikk.fhi.no/.

[75] Terveyden ja hyvinoinnin laitos (THL). Koronarokotteet [Internet]. thl.fi. 2025 [cited 2025 Jun 1]. Available from: https://thl.fi/aiheet/infektiotaudit-ja-rokotukset/rokotteet-a-o/koronarokotteet.

[76] folkhalsomyndighetebn. Statistik för influensavaccinationer [Internet]. folkhalsomyndigheten.se. [cited 2025 May 1]. Available from: https://www.folkhalsomyndigheten.se/folkhalsorapportering-statistik/statistikdatabaser-och-visualisering/vaccinationsstatistik/statistik-for-influensavaccinationer/.

[77] Hungnes O, Hessevik Paulsen T, Rohringer A, et al. Influenza Virological and Epidemiological 2023-2024 season report [Internet]. Norwegean Institute og Public Health (NIPH); 2024 [cited 2025 May 1]. Available from: https://www.fhi.no/contentassets/404d5692423743a68b346cbb2b7f8d99/final-2023-24-influenza-season-oct-2024_3.pdf?utm_.

[78] Terveyden ja hyvinoinnin laitos (THL). Influenssaepidemia jatkuu edelleen, rokotteen suojateho on kohtuullisen hyvä [Internet]. thl.fi. 2024 [cited 2025 May 1]. Available from: https://thl.fi/en/-/influenza-epidemic-continues-vaccine-protection-is-fairly-good?utm.

[79] Sundhedsdatastyrelsen. Medstat [Internet]. [cited 2025 May 13]. Available from: https://medstat.dk/.

[80] Finnish Medicines Agency Fimea. Finnish statistics on medicines 2023 [Internet]. 2023. Available from: https://www.julkari.fi/bitstream/handle/10024/150090/Finnish_statistics_on_medicines_2023.pdf?sequence=5&isAllowed=y.

[81] Socialstyrelsen. Statistikdatabas [Internet]. socialstyrelsen.se. [cited 2025 Jun 17]. Available from: https://www.socialstyrelsen.se/statistik-och-data/statistik/statistikdatabasen/.

[82] Direktoratet for medisinske produkter. Legemiddelsøk [Internet]. legemiddelsok.no. 2025. Available from: https://www.legemiddelsok.no/.

[83] Orjasniemi S, Palmén O. 2024 Ageing Report Finland - Country Fiche [Internet]. Ministry of Finance, Finland; 2023. Available from: https://economy-

finance.ec.europa.eu/document/download/31d1aa33-cf8b-4583-974a-51381cb6cb37_en?filename=2024-ageing-report-country-fiche-Finland.pdf.
